# Sensory and volatile compound profiles in tempeh-like products from faba bean and oats

**DOI:** 10.1016/j.crfs.2025.101029

**Published:** 2025-03-17

**Authors:** Laura Alejandra Fernandez Castaneda, Shania Saini, Oskar Laaksonen, Anna Kårlund, Su-lin L. Leong, William R. Newson, Volkmar Passoth, Kati Hanhineva, Maud Langton, Galia Zamaratskaia

**Affiliations:** a Department of Molecular Sciences BioCenter, Swedish University of Agricultural Sciences, SE-750 07, Uppsala, Sweden; b Food Sciences Unit, Department of Life Technologies University of Turku, FI-20014, Turku, Finland; c Department of Plant Breeding, Swedish University of Agriculture (SLU), 234 22, Lomma, Sweden

**Keywords:** Tempe, Tempeh, Plant-based, Lactic acid bacteria, Odour, Fermentation, Sensory evaluation

## Abstract

Faba beans (*Vicia faba* L.) and whole-grain oats (*Avena sativa* L.) offer a high protein content, are a source of dietary fibre and they can be sustainably produced in the Nordic countries, yet face sensory limitations that restrict their wider use in food. This study aimed to overcome these sensory limitations by soaking the faba bean and whole-grain oats with *Lactiplantibacillus plantarum*, followed by solid-state fermentation with *Rhizopus microsporus* to produce tempeh-like products. Volatile organic compounds in uncooked and cooked tempeh were analysed using Headspace Solid-Phase Micro-Extraction (HS-SPME) with Gas Chromatography-Mass Spectrometry (GC-MS). Sensory profiling was combined with texture and moisture measurements from uncooked and cooked tempeh-like products to explore sensory and physicochemical attributes. A hedonic test evaluated overall acceptability of the selected samples. A total of 65 volatile organic compounds were identified and semi-quantified, including 3-methyl-1-butanol, ethanol, 2-butanone, acetic acid, and acetoin. *L. plantarum* soaking reduced certain volatile organic compounds typically associated with beany off-flavours, while cooking increased certain compounds such as acids and pyrazines, potentially masking off-flavours. The soaking resulted in increased sourness, umami taste, and chewiness, with notable texture variation when whole-grain oats were included compared to only faba bean tempeh. Texture measurements were significantly influenced by *L. plantarum* soaking, as shown by the panellist. Hedonic testing (*n* = 107) indicated a higher degree of liking for tempeh made from the faba beans and whole-grain oats mix compared to only faba bean tempeh. These findings emphasise the need to assess how pre-treatments, such as soaking, impact on tempeh-like production.

## Highlights

• *L. plantarum* soaking enhanced sensory attributes of faba/oat tempeh.
• Fermentation increased umami and sour tastes, masking the off-flavours in faba bean.
• *L. plantarum* soaking increased chewiness and reduced graininess in faba/oat tempeh.
• Adding oat to faba bean tempeh resulted in higher overall liking.
• 65 volatile organic compounds identified in faba bean and whole-grain oat tempeh.

## Graphical abstract

**Figure:**
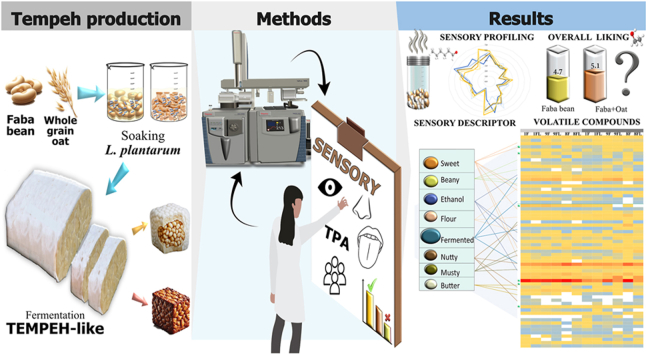

**Table:** 

Abbreviation	Description
SSF	Solid-state fermentation
1F	100 % faba bean, 0 % whole-grain oat, no *L. plantarum*
1FL	100 % faba bean, 0 % whole-grain oat, yes *L. plantarum*
8F	85 % faba bean, 15 % whole-grain oat, no *L. plantarum*
8FL	85 % faba bean, 15 % whole-grain oat, yes *L. plantarum*
9F	92 % faba bean, 8 % whole-grain oat, no *L. plantarum*
9FL	92 % faba bean, 8 % whole-grain oat, yes *L. plantarum*
C	Cooked
dw	Dry weight basis
GC-MS	Gas chromatography-mass spectrometry
GDA	Generic descriptive analysis
HS-SPME	Headspace solid-phase micro-extraction
*L. plantarum*	*Lactiplantibacillus plantarum*
LAB	Lactic acid bacteria
LSmeans	Least squares means
MEA	Malt extract agar
NaHCO_3_	Sodium bicarbonate
PCA	Principal component analysis
PCA	Principal component analysis
PLS	Partial least squares regression
R. microsporus	*Rhizopus microsporus*
SE	Standard error
TPA	Textural profile analysis
U	Uncooked
VOCs	Volatile organic compounds
w	Weight
ww	Wet weight basis

## 1 Introduction

Tempeh is a traditional Indonesian fermented food made from soybeans. The production process involves steps such as soaking, dehulling, boiling, and inoculating with *Rhizopus microsporus* for solid-state fermentation (SSF) (Ahnan‐Winarno et al., 2021), (Dinesh Babu et al., 2009). Tempeh provides a high protein content, carbohydrates, dietary fibre, and low levels of saturated fats (Romulo and Surya, 2021), (Sjamsuridzal et al., 2021).

Soybean tempeh is characterised by nutty, umami, mushroom-like, and buttery flavours, along with an entirely white-covered, compact appearance, and chewable texture by expert panellists from Indonesia (Jeleń et al., 2013). Tempeh sensory characteristics are highly influenced by the cooking method, with frying being the most popular. Steaming, roasting, and other preparation methods are also commonly used (Feng et al., 2007), (Kaczmarska et al., 2021). Its popularity in Europe as a plant-based meat alternative aligns with the planetary health diet, advocating for a more sustainable plant-based diet (Beal et al., 2023).

Nevertheless, soybean is considered a less sustainable raw material in Northern Europe due to its reliance on imports and consumer associations with deforestation, genetically modified organisms (GMOs), and allergenic properties (Fogelberg, 2021). Therefore, the development of non-soy tempeh has focused on utilising locally available legumes and cereals, which provide regional adaptability and a high nutritional profile due to the fermentation (Rahmawati et al., 2021). Faba beans and whole-grain oats could emerge as promising raw materials for tempeh production in colder climates (Auer et al., 2023). Faba beans provide high-quality protein, bioactive compounds such as L-DOPA and dietary fibre, offering potential benefits such as improved cardiac health and glycaemic control (Kadar et al., 2018), (Martineau-Côté et al., 2022). Similarly, meta-analyses show that whole-grain oats effectively lower total cholesterol (Marshall et al., 2020). Moreover, consumers exhibit a higher familiarity with oat-derived food products in the Nordic countries (Laaksonen et al., 2020). This may facilitate the enhancement of the sensory profile of tempeh from faba beans and oats, potentially improving its acceptance and overall appeal. The consumption of faba beans (*Vicia faba* L.) and their derived food products are constrained by undesirable sensory attributes such as bean-like flavours and bitter taste linked with specific volatile and non-volatile organic compounds. The high lipoxygenase activity in this kind of legume catalyses the oxidation of polyunsaturated fatty acids, resulting in volatiles like hexanal, 1-hexanol, and 2-pentylfuran that impart grassy, green, and earthy notes (Akkad et al., 2019), (Tuccillo et al., 2022a). Non-volatile organic compounds, such as phenolic compounds, and the degradation products of certain amino acids (e.g. leucine, alanine) have been linked to bitterness and trigeminal sensations such as astringency in faba bean flours (Akkad et al., 2019). Nevertheless, processing techniques such as soaking, boiling, and fermentation can alter the sensory profile by reducing the formation of off-flavours and/or masking them with desirable volatile compounds (He et al., 2022), (Tuccillo et al., 2022b).

Sensory characteristics of plant-based substrates can be enhanced by using an appropriate microbial consortium. For instance, fermentation with lactic acid bacteria, specifically *Lactiplantibacillus plantarum* (*L. plantarum*) in legumes and cereals improves the odour profile by reducing aldehydes and furans, such as hexanal and 2-furfural, which are associated with beany and grassy off-flavours (Roland et al., 2017). It also generates odour compounds that may mask these undesirable notes with sour and buttery notes such as acetic acid, ethanol, diacetyl, ethyl acetate, and acetaldehyde (Roland et al., 2017), (Tangyu et al., 2023).

In tempeh fermentation, SSF with *R. microsporus* contributes to sweet, mushroom-like, buttery, and meaty odours due to its proteolytic activity with the generation of 2,3-butanedione and 2-methylbutanal. *R. microsporus* promotes the formation of free amino acids and dipeptide flavour precursors, such as glutamyl dipeptides, within specific concentration ranges that enhance umami and savoury tastes. *R. microsporus* has been described to produce desirable compounds such as acetaldehyde, ethanol, 2-methylpropanal, and 2-propanol, while also reducing hexanal levels (Feng et al., 2007), (Xiao et al., 2024). In terms of texture, mycelial growth binds the beans into a cake-like structure, enhancing moisture retention and improving texture by increasing chewiness and compactness, while reducing the typical graininess and grittiness associated with beans (Handoyo and Morita, 2006), (Erkan et al., 2020). The use of microbial consortia has shown that *L. plantarum* does not hinder *R. microsporus* growth in tempeh (Feng et al., 2007), (Fernandez Castaneda et al., 2024). However, the effects of induced pre-fermentation during soaking with *L. plantarum* (LAB) on sensory attributes and volatile organic compounds (VOCs) in tempeh-like made using alternative raw materials remain unexplored.

Previous studies have not characterised the sensory properties of faba bean-oat tempeh, often masking inherent flavours through deep frying with oil. This study evaluates uncooked and cooked (no oil added) faba bean-oat tempeh. The impact of LAB pre-fermentation during soaking, followed by SSF with *R. microsporus*, on tempeh-like products were investigated. Three samples were tested using Nordic raw materials: faba bean alone and two faba bean-oat mixtures in different ratios. Soaking with and without LAB pre-fermentation was compared in both uncooked and cooked tempeh-like samples. The focus was on analysing volatile profiles, sensory attributes, and its likability using an affective test (hedonic scaling). The findings reveal the potential of traditional fermentation to create sustainable plant-based food from Nordic ingredients.

## 2 Materials and methods

### 2.1 Study materials

The faba beans (*Vicia faba* L. var. Gloria) were sourced from the Research Institutes of Sweden (RISE, Gothenburg, Sweden). Cultivated in central Sweden, the beans were harvested in 2020 and dehulled in 2021 (Hi-Tech Machinery Manufacturing Co., Ltd., Ningbo, China). Whole-grain oat (*Avena sativa* L.), hulled and steam-treated, were grown in Sweden and processed in 2021 (Lantmännen Oats AB, Kimstad, Sweden). Food grade sodium bicarbonate (NaHCO_3_) was bought from a supermarket in Uppsala, Sweden (Eldorado, Bohus, Sweden).

For pre-fermentation during the soaking process, *L. plantarum* (Harvest LB-1, pure culture, freeze-dried) provided by Novozymes (Bagsvaerd, Denmark), was used with a total cell count exceeding 10^11^ CFU/g, as specified by the manufacturer.

For SSF, *R. microsporus* in powdered form was obtained from TopCultures (Zoersel, Belgium). Powder was spread on malt extract agar (MEA) plates (Oxoid, Basingstoke, UK) and incubated at 30 °C in a Binder BF series convection incubator (class 3.1, Tuttlingen, Germany). After 6 days, the spores were harvested and subjected to four wash cycles using 9 g/L NaCl aqueous solution. The suspensions were centrifuged (Thermo Scientific Sorvall LYNX 4000, Waltham, MA, USA) at 5000×*g* for 10 min. The remaining spore suspension was mixed and its concentration was determined using a Bürker counting chamber (Hirschmann EM, Eberstadt, Germany). The suspension was stored at 4 °C (Beko model B5RCNA365HW, Istanbul, Türkiye) for a maximum of 2 days before use in tempeh production.

### 2.2 Tempeh-like products preparation

The lab-scale production of tempeh-like products was adapted from a method originally developed for faba beans, with modifications to include whole-grain oats in some samples, Fig. 1 (Fernandez Castaneda et al., 2024).

**Fig. 1:**
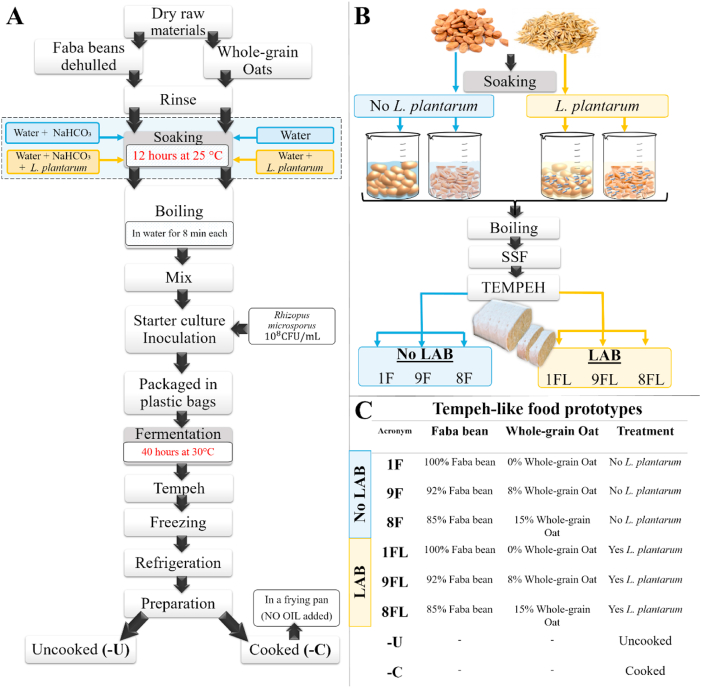
A. Flow chart depicting the lab-scale faba bean and whole-grain oat tempeh production processing line. B. Graphical flow chart in summary of lab-scale tempeh production. C. Description of food prototypes ingredients, soaking and cooking treatments. NaHCO_3_= Sodium bicarbonate.

Two treatments were studied in this experiment. The first treatment, referred to as "No LAB," involved soaking faba beans and whole-grain oats (separately) without the addition of *L. plantarum*. In this case, only tap water was used in a ratio of 1:2.5 (beans/oats:water) both raw materials on a dry weight basis. The second treatment, labelled as "LAB," involved adding 0.1 g of *L. plantarum* dissolved in tap water to soak 100 g of either faba beans or whole-grain oats. The soaking was carried out at a consistent ratio of 1:2.5 (beans/oats:water), with both raw materials measured on a dry weight basis.

Six samples were prepared, consisting of three prototypes, all based in wet weight: 100 % faba bean only; 92 % faba bean +8 % whole-grain oat and 85 % faba bean +15 % whole-grain oat, which included each prototype from soaking treatment without and with *L. plantarum* (Fig. 1C). Additionally, NaHCO_3_ (1 % w/dw of beans) was used in faba bean soaking in all treatments to reduce boiling time (Fernandez Castaneda et al., 2024).

After soaking for 12 h at 25 °C, the beans and oats were rinsed thoroughly and boiled in water separately for 8 min.

After soaking and boiling, faba bean and whole-grain oat were allowed to cool down to room temperature; then 1 mL of spore suspension of *R. microsporus* per 100 g wet raw material was added (approximately 10^8^ CFU/mL), mixed, and tightly packed into perforated food-grade zip-lock plastic bags to facilitate oxygen exchange. The packaging dimensions were 100 × 150 × 0.05 mm (length, width, and thickness) using LDPE food-grade material (manufactured in China and distributed by VWR, Leuven, Belgium). Tempeh-like products had dimensions of 100 × 150 × 25 m. Fermentation process was conducted for 40 h at 30 °C.

Tempeh samples were stored at −20 °C until analysis. The frozen samples were shipped to the University of Turku, Finland and stored at −20 °C until analysis (Beko model B5RCNA365HW, Istanbul, Türkiye). Tempeh samples used for the affective test in Sweden were newly prepared and stored at −20 °C.

### 2.3 Sample preparation for analysis

The samples were thawed at 4 °C (Beko model B5RCNA365HW, Istanbul, Türkiye) for 24 h prior to sensory profiling with the semi-trained panel. The samples were cut into rectangles of 3 × 2 × 1 cm (length, width, and thickness). The frying pan was preheated for 3 min at a temperature setting of 7 out of 10 on a conduction stove (Bosch, Stuttgart, Germany). The tempeh samples were then roasted for 2 min on each side. All samples were cooked under the same conditions. Although tempeh can be prepared in various ways, deep frying in seed oil is the most common method. However, in this study, no oil was used to avoid any potential flavour alterations caused by the oil. The same sample preparation was performed for analysis of volatiles compounds, sensory study, texture profile, and affective test (hedonic scale).

### 2.4 Analysis of volatile organic compounds (VOCs) by HS-SPME-GC-MS

For HS-SPME, 2.0 g of uncooked and cooked tempeh samples were placed separately in 20 mL SPME glass vials and incubated for 10 min at 50 °C. A 2 cm DVB/CAR/PDMS fibre (50/30 μm, Supelco, Bellefonte, PA, USA) was used for extraction of volatile organic compounds from the vial headspace. The fibre was pre-conditioned for 1 min and post-conditioned for 7 min at 240 °C. Each sample was analysed in triplicates. Next, the fibre with the extracted volatile organic compounds was transferred to the Trace 1300/1310 gas chromatograph, coupled with an ISQ 7000 single quadrupole mass spectrometer (Thermo Fisher Scientific, Waltham, MA, USA). The sample vial parameters included a needle speed of 20 mm/s, an extraction time of 30 min, a sampling vial depth of 45 mm, an injection depth of 40 mm, a penetration speed of 40 mm/s, and a desorption time of 5 min.

Separation was carried out using a DB-WAX polar capillary column (60 m length × 0.25 mm i.d. × 0.25 μm film thickness, J&W Scientific, Folsom, CA, USA). Helium was the carrier gas used, at a flow rate of 1.6 mL/min and in split-less mode. The gas chromatograph oven was programmed to start at 40 °C for 3 min, followed by an increase of 5 °C/min to a final temperature of 200 °C, with a holding time of 8 min. The temperatures of the MS transfer line and the ionisation source were approximately 220 °C and 240 °C, respectively. Peak integration was performed manually, and compound identification was achieved by comparing the MS spectra with the NIST_14_ database (National Institute of Standards and Technology), using the Chromeleon 7.2.9 Chromatography Data System (Thermo Fisher Scientific, Waltham, MA, USA). The matching factors (MFs) were set to be > 800. The results are reported as peak areas (counts × s × 10^6^).

### 2.5 Sensory study

#### 2.5.1 Ethical approval

The sensory evaluation on tempeh-like samples was approved by the Ethics Committee for Human Sciences, Humanities and Social Sciences Division, University of Turku, Finland, Case Number TY/138/06.January 01, 2024, granted on 01-03-2024. Each participant provided written consent prior to their participation in the study. The privacy notice was disclosed according to Articles 13 and 14 of the European Union (EU) General Data Protection Regulation and no monetary compensation or incentives were given.

#### 2.5.2 Panels

The semi-trained panel was recruited via email and consisted of eight healthy volunteers from the Food Sciences unit at the University of Turku, all with a background in food science and prior experience in sensory evaluation and quantitative methods, ensuring familiarity with sensory attributes relevant to plant-based products. Training sessions included calibration exercises with reference compounds, focusing on key aroma, taste, and texture descriptors. Panellists practiced intensity scaling to ensure consistency and reliability in evaluations. They participated in all training and evaluation sessions.

The hedonic panel included 107 healthy volunteers (ages 23–70), comprising students and staff from the University of Turku in Finland and the Swedish University of Agricultural Sciences in Sweden. Recruited through public advertisements and university mailing lists, participants provided informed consent and evaluated overall sensory attributes using a structured hedonic scale. The panel featured diverse demographics with a broad age range (23–70) and was selected based on practical access to university facilities. Conducting the study across two countries (Finland and Sweden) added cross-cultural insights.

#### 2.5.3 Panel training and generic descriptive analysis (GDA) sensory evaluation

The training process was conducted over three sessions on different days, each lasting 2 h. The training process began with a generic descriptive analysis, during which numerous attributes were listed. The selection of reference materials was based on previous sensory studies of faba bean ingredients with modification (Tuccillo et al., 2022a). The panellists were familiarised and trained using the reference samples to ensure precision in evaluating each attribute.

Standard reference samples for the descriptors were used for the sensory profiling of tempeh-like products (Appendix A1 Table A1). All samples were cooked in a frying pan under identical conditions as previously described. Herein, a commercial faba bean tempeh (GreenJava®, Lieto, Finland) was used as reference product alongside two of the developed products during training sections.

The descriptors and intensity levels were decided by the panel after joint discussions with the panel leader (Table A1). For the final descriptive test, sensory profiling was conducted by a semi-trained panel (n = 8) with test samples evaluated as triplicates on three consecutive days. All tempeh samples were cut into 3 × 2 × 1 cm (length, width, and thickness) and cooked in a frying pan under the same conditions as previously described with no oil addition in section 2.3.

The tests were divided into two and evaluated using intensity line scales (0 as zero intensity–10 as maximum intensity). In the first tests, the uncooked samples (Fig. 1C) were served in brown amber bottles, and their odour attributes were evaluated. In the second tests, the odour, appearance, taste, and texture of cooked samples were evaluated. These samples were served in glass bowls covered with Petri plates, labelled with randomised three-digit codes, and presented in a balanced partially randomised order (Williams Design model). Water, crackers (for tastes), and ground coffee (for odours) were served for palate cleansing to conform to the laboratory requirements set under ISO 8589. The sensory tests were performed with Compusense20 (version 23; Compusense Inc., Guelph, ON, Canada).

#### 2.5.4 Affective test (hedonic scaling)

A group of non-trained panellists (n = 107 volunteers) participated in the evaluation of three tempeh samples (cooked), all made with the addition of *LAB* in the soaking process. The samples were 1FL, 9FL and 8FL (Fig. 1C). All tempeh samples were cut into 3 × 2 × 1 cm (length, width, and thickness) and cooked in a frying pan under the same conditions as previously described with no oil addition in section 2.3.

Participants were asked to taste the samples and then indicate their preference using a nine-point balanced hedonic scale with 1 = "Dislike extremely" and 9 = "Like extremely" for the overall acceptance of tempeh samples (questionnaire in Appendix A3.2). Samples were coded with three-digit numbers, and the evaluation order was randomised using Compusense20 (version 23; Compusense Inc., Guelph, ON, Canada).

### 2.6 Texture profile

A Texture Analyser (Stable Micro Systems Ltd., Surrey, UK) was used to conduct the Texture profile analysis (TPA) in cooked tempeh samples (Fernandez Castaneda et al., 2024). A double compression model was used to simulate chewing. After thawing at room temperature, the samples were cooked as previously described in section 2.3. Then, tempeh-like products were cut into 3 × 2 × 1 cm (length, width, and thickness) and compressed to 50 % of their original height using a 20 mm diameter cylindrical acrylic probe. The pre-test speed was 2.0 mm/s, with the test and post-test speeds at 5.0 mm/s, and a 5-s interval between compressions. A 50 kg load cell measured the applied force. Key texture attributes were analysed, including hardness (peak force during the first compression), adhesiveness (area under the curve for the first negative peak), cohesiveness (ratio of areas under the second and first compression curves), and chewiness (product of hardness, cohesiveness, and springiness). The software version TA.XT plus C (Stable Micro Systems, TA-HDi, Surrey, UK) was used. Three measurements were performed per sample from three different batches.

### 2.7 Moisture content

The moisture content of all uncooked and cooked tempeh-like samples was measured in triplicates by calculating the weight difference before and after drying overnight in an oven (Model, 2000655, J.P. Selecta, Barcelona, Spain) at 105 °C, following the AOAC official method 934.01.

### 2.8 Data analysis

Analysis of the effects of soaking and prototype on VOCs, TPA and sensory parameters was performed using SAS software, version 9.4 (SAS Institute Inc., Cary, NC, USA). A generalised linear model (GLM) included fixed factor of treatment soaking (with and without LAB), prototypes (Fig. 1), and their interaction (soaking∗prototype). Concentrations of VOCs, except for phenol, were log-transformed to normalise the distributions prior to analysis. Concentrations of phenol, TPA values, and sensory parameters were approximately normally distributed. All results were expressed as least squares means (LSmeans). The heat map (Fig. 2) visualises VOCs distribution across uncooked (-U) and cooked (-C) tempeh, with or without LAB. The colour scale reflects the mean values after statistical analysis, ranging from white to red, indicating the intensity of VOCs. VOCs LSmeans with 95 % confidence intervals (CI) were reported after back-transformation to the original scale (Table 1 and Appendix A2 Table A2). Analyses were conducted separately for uncooked and cooked samples. These data are presented with standard error (SE) and *p-values* for the treatments described above. For PCA, X-data included VOCs (n = 65) from uncooked and cooked samples, and sensory attributes (n = 21) from cooked samples. For PLS-regression, interactions were analysed between VOCs, TPA, and moisture content as predictors (X-data; n = 71) and sensory scores (Y-data; n = 21) in cooked samples. Statistical analyses for the multivariate models were performed using SIMCA® version 18, with p < 0.05 as the significance threshold (Appendix A5 Fig. 2, Fig. 3, Fig. 4). For the affective test, the hedonic scores were analysed using one-way ANOVA, followed by Tukey HSD for pairwise comparisons when significance was found (*p < 0.05*) performed using RStudio version 4.3.2 (RStudio Inc., Boston, MA, USA).

**Table 1:**
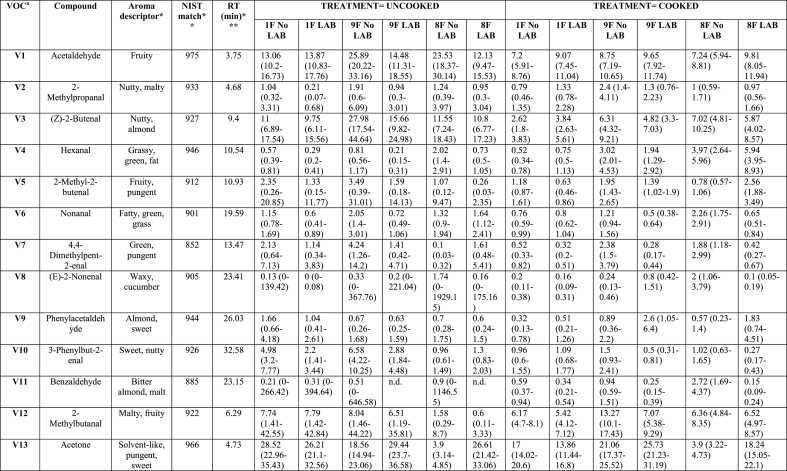

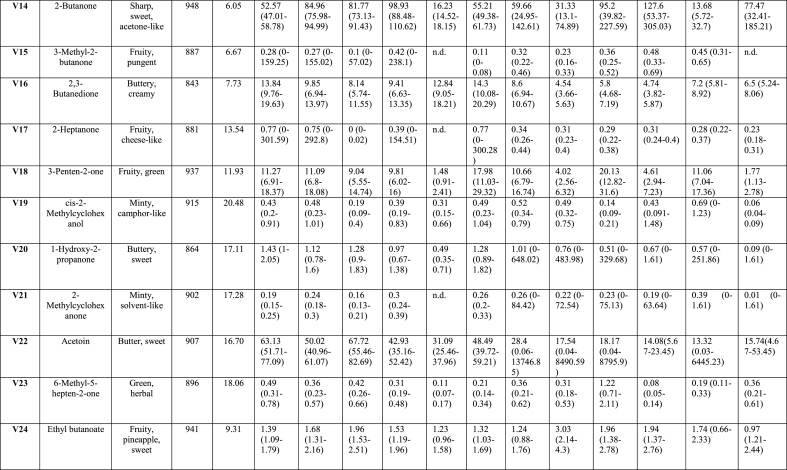

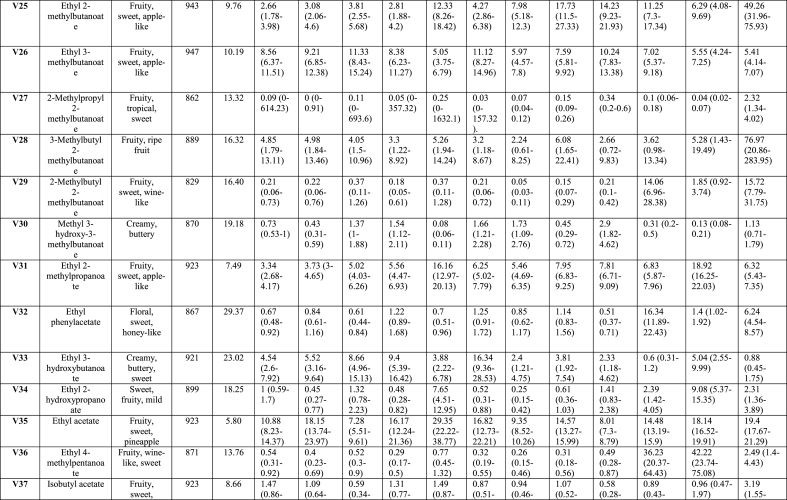

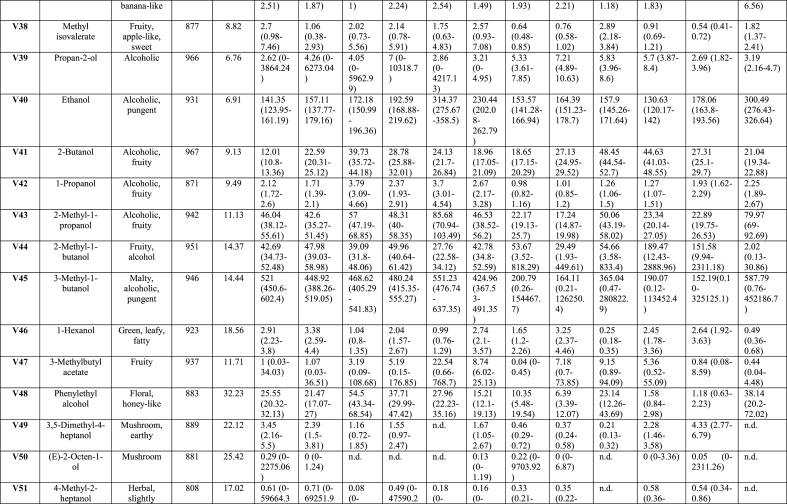

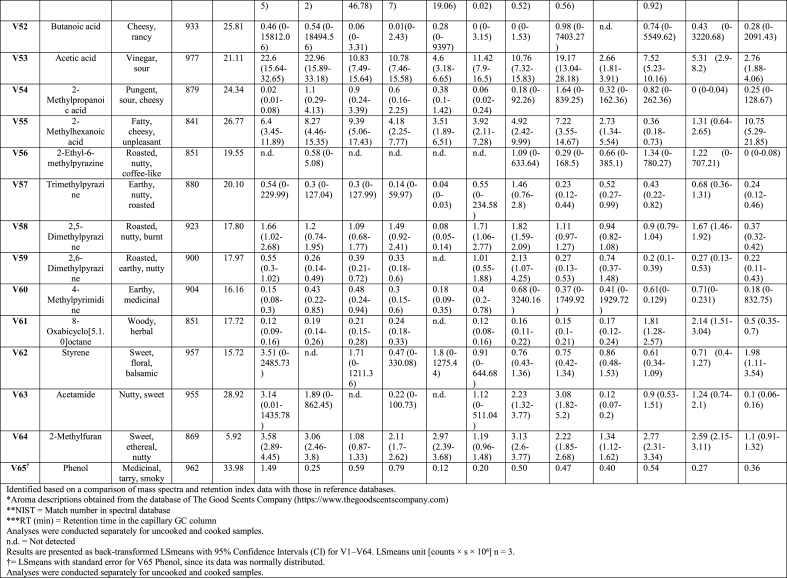
Volatile organic compounds semi-quantified and their main descriptors detected by HS-SPME-GC-MS of tempeh-like products made from faba bean and a mix of faba bean and whole-grain oat, pre-treated by soaking without or with L. *plantarum*, in uncooked and cooked states. Results are presented as LSmeans with 95 % Confidence Intervals (CI).

**Fig. 2:**
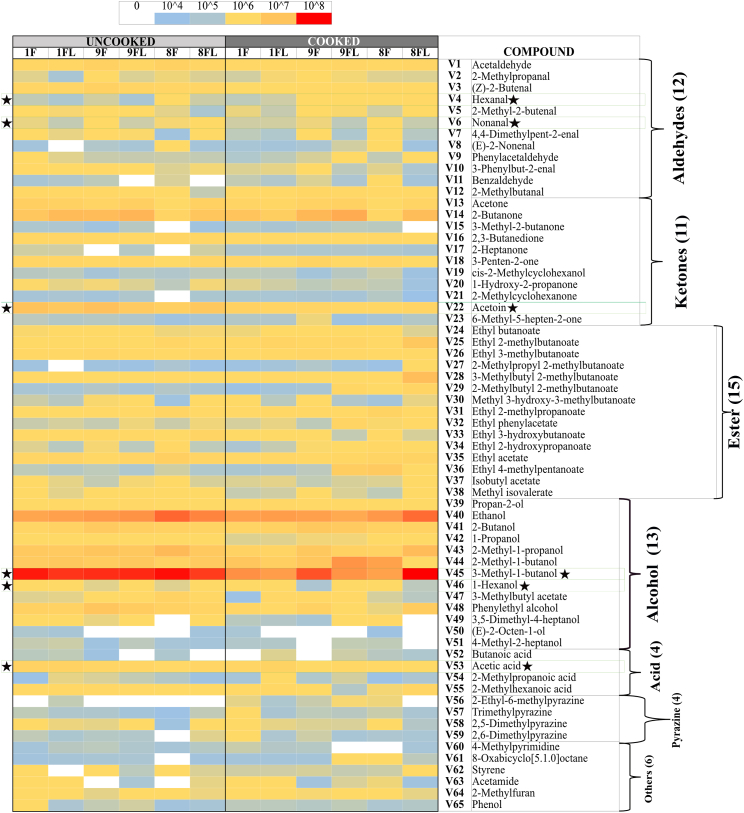
Heat map analysis for the discriminatory VOCs on the two main groups of –U: uncooked and –C: cooked, -No LAB and -LAB; VOC names are depicted on the right side and grouped by functional groups. Mark indicates some of the most influential VOCs in the flavour of tempeh-like products based on their well described sensory profile in food products; descriptions obtained from database of The Good Scents Company (https://www.thegoodscentscompany.com). Scale intensity is presented as the values of areas under the peaks (counts × s), colour ranging from white as zero to red as >10^8^ (counts × s).

**Fig. 3:**
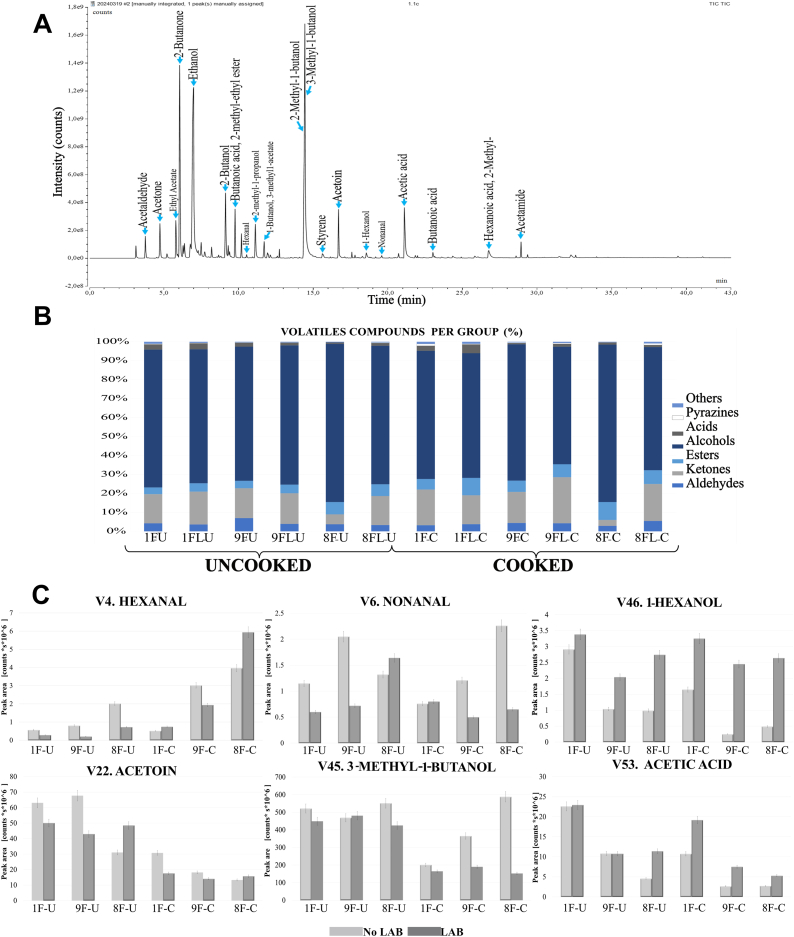
A. Typical chromatogram with the volatile organic compound profile of cooked 100 % faba bean tempeh with LAB as pre-treatment (1FL-C) with up to 43 min retention time. B. Category distribution ( %) of the VOCs by chemical group in the six tempeh-like products with uncooked and cooked samples. C. Selected VOCs associated with beany off-flavours hexanal, nonanal, and 1-hexanol, and selected desirable odour-masking compounds, acetoin, 3-methly-1-butanol, and acetic acid, in tempeh-like products, X-axis show the samples, Y-axis indicates the relative peak area intensity [counts∗s], n = 3. –U: uncooked and –C: cooked, light colours No LAB, dark colours LAB.

**Fig. 4:**
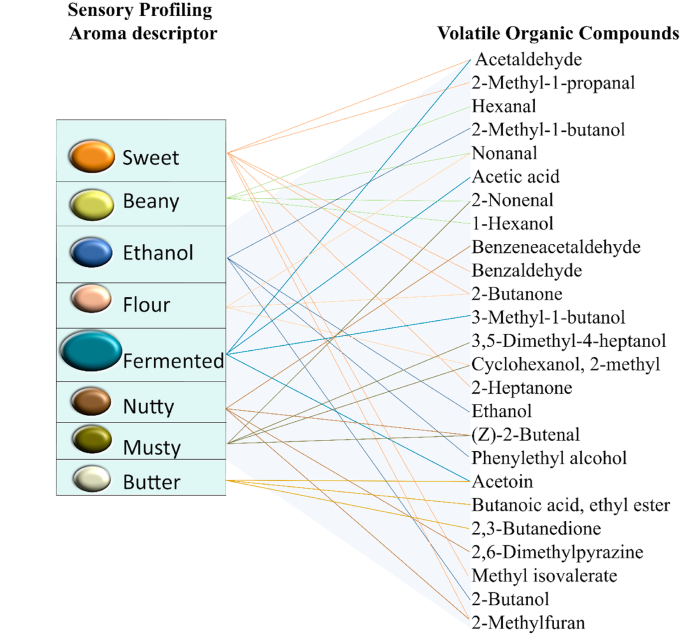
Correlation of dominant volatile compounds, based on their semi-quantification, with aroma descriptors obtained from The Good Scents Company database (https://www.thegoodscentscompany.com).

## 3 Results and discussion

### 3.1 Analysis of volatile organic compounds (VOCs)

#### 3.1.1 Identification of signals

VOCs were analysed using GC–MS combined with HS–SPME to explore, in a semi-quantitative manner, the variations in volatile profiles among the tempeh-like products. Six tempeh-like products were tested in two forms: uncooked and cooked (Fig. 1C). The objective was to assess changes due to raw materials (prototypes) and soaking pre-treatment, categorised as No LAB (without *L. plantarum*) and LAB (with *L. plantarum*), as well as the interaction effects. Roasting in a frying pan for 2 min per side was selected as the cooking method and applied under the same conditions as in the sensory profiling, physical properties, and volatile tests.

A total of 65 peaks were identified as VOCs in tempeh-like products (Table 1) and odour descriptors were sourced from **The Good Scents Company (**Oak Creek, WI, USA). Some of the dominant volatiles detected in both uncooked and cooked faba/oat tempeh were 3-methyl-1-butanol, ethanol, 2-methyl-1-butanol, 2-butanol, and 2-methyl-1-propanol, which primarily originate from tempeh fermentation with *R. microspores* (Feng et al., 2007). In contrast, 2-butanone, acetoin, acetic acid, and ethyl acetate may be produced through LAB fermentation in the soaking (Tangyu et al., 2023), (Engels et al., 2022). (Appendix A2 Table A2 statistical analysis results *p-values*). VOCs were categorised into seven chemical groups: 15 esters, 13 alcohols, 12 aldehydes, 11 ketones, 4 acids, 4 pyrazines, and 6 others (Fig. 2, Fig. 3).

#### 3.1.2 Analysis per chemical group of VOCs

The total composition per chemical group, expressed as total percentages, highlights the differences between uncooked and cooked samples (Fig. 3B), as well as between No LAB and LAB. Significant differences (*p < 0.05*) were observed in the total concentration of VOCs per chemicals group. There was a tendency for increased levels of ketones, esters, acids, and pyrazines in the cooked samples, along with a slight increase in compounds from other chemical composition groups. In contrast, a significant decrease (*p < 0.05*) in the intensity of alcohols was observed in the cooked samples. Aldehydes showed a varied trend, with some samples exhibiting a slight decrease, while others showed an increase after cooking. Moreover, the soaking process with LAB resulted in increased esters and acids, while also leading to slightly higher pyrazine levels compared to No LAB-soaked tempeh-like products. In contrast, a lower intensity of alcohols and aldehydes was observed in the LAB-treated samples (Fig. 3B).

Of the fifteen esters identified, none were dominant due to their lower sensory detection thresholds compared to alcohols and acids. These esters were formed through reactions between carboxylic acids with alcohols through coenzyme A (CoA) (Rajendran et al., 2023). Despite a low abundance, their sweet and fruity notes created distinct sensory perception for each sample, significantly influencing (*p < 0.05*) the sensory profile. As an example, ethyl acetate, which has been considered a desirable VOC in fermented legumes (Vong and Liu, 2017), was detected in all samples in present study (Fig. 2, V35). This compound was likely developed from the SSF with *R. microsporus*, as detected in other studies with MEA agar, barley, soybean and sweet potato residues (Feng et al., 2007) (Yin et al., 2023). Moreover, an increase of ethyl acetate in barley tempeh was found in co-cultivation with *R. microsporus* and *L. plantarum* SR 3.60 (Feng et al., 2007). In this study, in samples tempeh 100 % faba bean-LAB (1FL) and 92 % faba bean + 8 % whole-grain oat LAB (9FL) the VOC ethyl acetate showed higher levels than in 100 % faba bean-No LAB (1F) and 92 % faba bean + 8 % whole-grain oat-No LAB (9F) sample. Therefore, there is a positive correlation between the presences of *L. plantarum* and an increase in ethyl acetate. However, in 85 % faba bean +15 % whole-grain oat No LAB (8F) samples, high concentrations of ethyl acetate were found in both, the uncooked and cooked groups.

Thirteen alcohols were detected in tempeh-like products produced during this study. These included most dominant compounds such as 3-methyl-1-butanol, ethanol, 2-methyl-1-propanol, 2-methyl-1-butanol, and 2-butanol, based on their high values of areas under the peaks (counts × s × 10^6^). Among these, 3-methyl-1-butanol (V45) was the most dominant VOC across all samples. Previous studies have also reported 3-methyl-1-butanol as a dominant compound in tempeh (Feng et al., 2007), (Chukeatirote et al., 2017). This alcohol is a product of leucine amino acid catabolism and imparts malty and alcoholic notes but it can be pungent and unpleasant at high concentrations (Rajendran et al., 2023). Some LAB strains, for instance, strains of *L. plantarum* and *L. acidophilus* have been shown to reduce its levels in plant-based materials (Rajendran et al., 2023). For this case, soaking pre-treatment with LAB in combination with cooking further decreased 3-methyl-1-butanol VOC significantly (*p < 0.05*). Notably, the 8F samples in U and C (No LAB) had the highest levels of 3-methyl-1-butanol, as well as ethanol and 2-methyl-1-propanol. Interestingly, in 8F samples (U and C), other alcohols compounds such as 4-heptanol, 3,5-dimethyl-; (E)-2-octen-1-ol; and 4-methyl-2-heptanol were not detected. The high content of whole-grain oat may be responsible for the significant changes, likely affecting carbohydrate fermentation pathways and amino acid catabolism, leading to varied alcohol production.

1-Hexanol, a key contributor of beany off-flavour which is produced via enzymatic oxidation of linoleic acid and is commonly found in the VOC profiles of legumes and cereals, has been quantified in faba beans without fermentation (Tuccillo et al., 2022a). A significant increase (*p < 0.05*) in 1-hexanol was observed in LAB treated samples (Fig. 3C), suggesting its formation was primarily due to auto-oxidation of polyunsaturated fats. The sample with No LAB had significantly lower (*p < 0.05*) levels of 1-hexanol in the cooked group but it was not the case for LAB group.

In this study, (E)-2-octen-1-ol, was detected in five out twelve samples (Table 2, Fig. 2 V50). For instance, the highest levels were found in 1F uncooked and cooked, while a slight increase was found in the group with LAB. According to Chukeatirote et al., (E)-2-octen-1-ol was detected only in tempeh from soybean fermented in co-cultivation of *R. microsporus* and *Bacillus subtilis* TN51 (Chukeatirote et al., 2017). This compound is rarely mentioned in plant-based fermented foods but is well-characterised in edible mushrooms like pine mushroom (*Tricholoma matsutake* (S. Ito and S. Imai) Singer), where it contributes to the desirable mushroom flavours with distinct fungal and buttery notes (Cho et al., 2008).

**Table 2:** Textural and moisture properties of tempeh-like products made from faba bean and a mix of faba bean and whole-grain oat, pre-treated by soaking without or with *L. plantarum*, in the uncooked and cooked states.

Prototype	1F 100 % faba bean		9F 92 %Faba bean+8 %whole-grain oat		8F 85 %Faba bean+15 %whole-grain oat		SE	*p-value*, Prototype	*p-value*, LAB	*p-value*, Prototype∗LAB
Soaking	No LAB	LAB	No LAB	LAB	No LAB	LAB				
UNCOOKED										
Moisture ( %)	68.9^a^	68.3 ^a^	69.9^a^	67.9^a^	68.6^a^	65.7^a^	2.24	0.308	0.086	0.63
TPA										
Hardness (N)	23.0^d^	48.8^b^	34.2^c^	63.8^a^	55.2^b^	74.0^a^	2.24	**<0.001**	**<0.001**	0.089
Adhesiveness (N.s)	−1.3^a^	−1.3^a^	−1.9^a^	−0.^8a^	−0.8^a^	−1.7^a^	0.43	0.9703	0.795	0.0889
Cohesiveness	0.2^c^	0.3^ab^	0.2^c^	0.3^ab^	0.3^a^	0.3^a^	0.02	0.0902	**<0.001**	**0.001**
Springiness ( %)	33.0^c^	49.3^a^	25.8^b^	41.1^c^	36.8^c^	38.5^c^	2.20	**0.015**	**<0.001**	**0.010**
Chewiness (N)	1.5^d^	8.2^b^	2.0^d^	8.8^a^	6.3^c^	8.8^a^	0.48	**<0.001**	**<0.001**	**0.001**
COOKED										
Moisture ( %)	65.6^a^	63.2^a^	65.8^a^	63.7^a^	65.7^a^	66.2^a^	0.83	0.193	0.076	0.207
TPA										
Hardness (N)	56.3^c^	56.8^c^	51.9^d^	63.3^b^	73.5^a^	47.6^d^	2.1	0.197	**0.020**	**<0.001**
Adhesiveness (N.s)	−2.3^a^	−0.8^a^	−1.1^a^	−1.2^a^	−0.8^a^	−1.5^a^	0.44	0.504	0.520	0.070
Cohesiveness	0.3	0.3	0.3	0.3	0.3	0.3	0.016	0.953	0.424	0.051
Springiness ( %)	33.3^a^	34.5^a^	37.9^a^	35.1^a^	36.7^a^	40.6^a^	2.2	0.145	0.678	0.364
Chewiness (N)	6.2^c^	5.3^e^	5.5^d^	6.9^b^	8.0^a^	5.7^d^	0.25	**0.003**	**0.013**	**<0.001**

Values represent LSmeans and SE.

Mean values in the same row with different letters differ significantly (*p < 0.05*).

Bold *p-values* represent significant differences.

Moisture ( %) and TPA, n = 3. –U: uncooked and –C: cooked groups.

In the aldehydes group, 12 VOCs were detected in all samples. These VOCs are generated from various substrates such as amino acids, free fatty acids, and sugars (Roland et al., 2017). Notably, two important VOCs hexanal (V4) and nonanal (V6) were detected in all samples, as they have a significant negative influence on the sensory perception due to their beany, grass-like, fresh green, waxy, and earthy notes (Jiang et al., 2016). Hexanal is produced from the oxidation of linoleic acid, while nonanal is derived from oleic acid oxidation (Lampi et al., 2015).

Samples pre-treated with LAB contained of lower hexanal levels; while, only sample 8FL-C exhibited a significantly increased hexanal formation (*p < 0.05*) in cooked compared to uncooked. Conversely, while hexanal levels were the highest in the sample 8FL-C, nonanal showed the highest value in 8F-C without LAB. Both hexanal and nonanal were detected at low levels in 100 % faba bean tempeh (1F and 1FL) compared to faba bean-whole-grain oat tempeh, likely due to endogenous lipid oxidation and/or oxidation from the oats during cooking.

Hexanal and nonanal tended to slightly increase in samples treated with the combination of LAB and cooking; Feng, Larsen, and Schnürer (Feng et al., 2007) had reported hexanal and nonanal VOCs in soybean tempeh. However, in their study a total reduction of hexanal was observed after 20 h of SSF in barley tempeh with and without *L. plantarum* SR 3.60 (Feng et al., 2007); it was attributed to the action of SSF with *R. microsporus* (Feng et al., 2007).

Pentanal was not detected in this study, despite being frequently reported in faba beans and their fractions, where it negatively contributes to the sensory profile with a strong beany off-flavour (Akkad et al., 2019), (Tuccillo et al., 2022a), (Stone et al., 2024), (Xiang et al., 2023).

Some aldehydes are associated with desirable flavours, such as acetaldehyde with fruity notes, benzaldehyde with almond-like notes, and (Z)-2-butenal with roasted, nutty, and umami-like notes. All of these VOCs were detected in almost all tempeh samples, except for the samples 9FL and 8FL, where benzaldehyde was detected only in cooked samples (Fig. 2, V11). This component has been linked to lactic acid fermentation from the amino acid phenylalanine (Rajendran et al., 2023) and typical odour-active Maillard reaction end products including Strecker aldehydes (Li et al., 2022).

Eleven volatile ketones were detected in this study, with 2-butanone, acetoin, and acetone being the most dominant compounds in all tempeh samples, showing the highest peak areas within the ketone chemical group. The presence of 2-butanone in this study is associated with LAB, as most samples showed an increase in this compound, particularly, when combined with cooking. This ketone is perceived with sweet and solvent-like notes (Rajendran et al., 2023) and has been reported in another study as the most dominant compound in soybean tempeh, though it was also present in barley tempeh without and with LAB (Jeleń et al., 2013). Moreover, Stone et al. (2024), reported 2-butanone as one of the major volatile organic compounds in fermented chickpea and faba bean protein isolates, following fermentation with LAB, *Aspergillus oryzae* and *Aspergillus niger,* separately.

Acetoin (3-hydroxy-2-butanone) was consistently detected with high levels in all uncooked samples but significantly decreased (*p < 0.05*) in all cooked samples. Its presence in uncooked faba bean, whole-grain oat, and tempeh products has not been previously documented but Feng, Larsen, and Schnürer (Feng et al., 2007) noted its formation in MEA from *Rhizopus oligosporus* fermentation. Acetoin is perceived with desirable flavours of creamy and buttery notes (Xiao and Lu, 2014). A study found that LAB 23169 increased acetoin levels in mung beans, enhancing fermentation through its “deodorisation” effect and the development of other desirable VOCs (Yi et al., 2021).

In the 8F-U sample (No LAB), three VOCs as 3-methyl-2-butanone, 2-heptanone, and 2-methylcyclohexanone were not detected but they were present in the cooked sample. In contrast, 8FL-U showed detectable levels of these compounds, although in lower concentrations compared to other samples. Those compounds may play a flavour enhancing role due to their odour complexity, contributing fruity, sweet, and herbal notes, as found in mature cheeses (Engels et al., 2022).

Four acid VOCs were detected in all samples, with 2-methyl hexanoic acid, and acetic acid being the most dominant within the group. Hexanoic acid provides cheesy notes, while acetic acid gives sour and vinegar-like notes (Rajendran et al., 2023). In this study, acetic acid increased significantly (*p < 0.05*) in LAB samples, while comparing 1F and 1FL both in U and C treatments. The combined effect of *L. plantarum* and cooking intensified acetic acid (Fig. 3C, V53). Acetic acid is considered as an effective flavour-masking compound and has been reported in soybean tempeh and other fermented faba bean products (Feng et al., 2007), (Tuccillo et al., 2022b). However, its presence in tempeh may come from the direct addition of vinegar in the production process, as in the soaking medium and/or before *R. microsporus* inoculation (Feng et al., 2007). In our case here, no vinegar was added.

The concentration of pyrazines significantly increased (*p < 0.05*) with cooking due to Maillard reactions that were triggered by high-temperature exposure (Wang et al., 2021). In this study, VOCs such as 2-ethyl-6-methylpyrazine, 2,6-dimethylpyrazine, and two other related VOCs were identified in tempeh samples. These compounds are associated with nutty, caramelised, and roasted notes, which help to mask off-flavours in plant-based materials (Bi et al., 2020). For instance, 2-ethyl-6-methylpyrazine was detected in only 1 of the 6 uncooked samples (1FL-U). However, after cooking, it was present in all samples, where 9FL-U (uncooked) showed no detection, while 9FL-C (cooked) exhibited the highest concentration among all samples.

Six VOCs were classified in the “others” group, including ethers, amide, and furan. The most dominant compounds in this group were 2-methylfuran and phenol. 2-methylfuran increased in samples treated with LAB and after cooking. 2-methylfuran is a key VOC commonly found in soybean paste fermented with ***Aspergillus oryzae*** and in other brine fermentation processes on plant-based materials (Kum et al., 2015).

LAB soaking significantly increased certain esters, acids, and pyrazines, enhancing fruity, sour, and roasted notes while reducing or preventing the formation of high concentrations of alcohols and aldehydes, which contribute to beany and grassy off-flavours. LAB helped prevent excessive alcohol and aldehyde formation while promoting compounds that enriched the tempeh-like aroma, creating a more complex and improved flavour profile. Cooking further intensified roasted and nutty notes by increasing pyrazines and ketones while reducing alcohols and acetoin.

Comparing the different prototypes, the addition of whole-grain oats generally resulted in higher concentrations of ketones and aldehydes and lower alcohol levels, suggesting a more diverse aromatic profile, especially in cooked samples. The LAB soaking process had a significant impact on VOC concentrations in samples with whole-grain oats (Fig. 3B), indicating a richer profile that may enhance the tempeh-like flavour while also helping to mask off-flavours in faba beans and whole-grain oats.

#### 3.1.3 Impact of LAB soaking pre-treatment and SSF on Tempeh's VOC profile

This study demonstrated that soaking with *L. plantarum* as a pre-treatment altered the VOC profile of tempeh uncooked and cooked, where 32 of 65 VOCs (uncooked group) and 44 of 65 VOCs (cooked) were significantly different due to the “LAB” as explanatory/independent variable (no *L. plantarum* and *L. plantarum*) in the statistical model (Appendix A2 Table A2). *L. plantarum* treatment appeared to enhance the tempeh odour profile, through complex microbial and enzymatic processes. The mechanism of odour and flavour development may rely on the induced fermentation stage during the soaking process, where the production of lactic acid, acetic acid, ethanol, and carbon dioxide by *L. plantarum* leads to favourable changes in the VOC profile (Engels et al., 2022), (Yi et al., 2021). This metabolic shift resulted in increased levels of desirable VOCs as ketones, acids, and pyrazines, associated with sweet, buttery, nutty, and roasted notes, alongside simultaneously reduced formation of off-flavours, typically associated with some alcohols and aldehydes mentioned previously (Rajendran et al., 2023).

These sensory improvements were further amplified during SSF with *R. microsporus* in the tempeh production process as it generated proteases, lipases, and amylases, which broke down proteins, fats, and starches into VOCs, enriching the aromatic profile with malty, nutty, fruity, and alcoholic notes. For instance, *R. microsporus* produces pectinases and cellulases, releasing sugars that participate in Maillard reactions and caramelisation in heat exposure, leading to the formation of pyrazines that impart roasted, nutty, and sweet notes (Yin et al., 2023). Fig. 4 shows correlation between some of the most dominant and significant VOCs with aromatic descriptors.

However, understanding the flavour of fermented legume-cereal products requires linking VOC analysis to sensory perception. The next section uses descriptive analysis to explore how volatile compounds shape the sensory attributes, highlighting key odour and taste characteristics.

### 3.2 Sensory evaluation

#### 3.2.1 Descriptive analysis

**Fig. 5:**
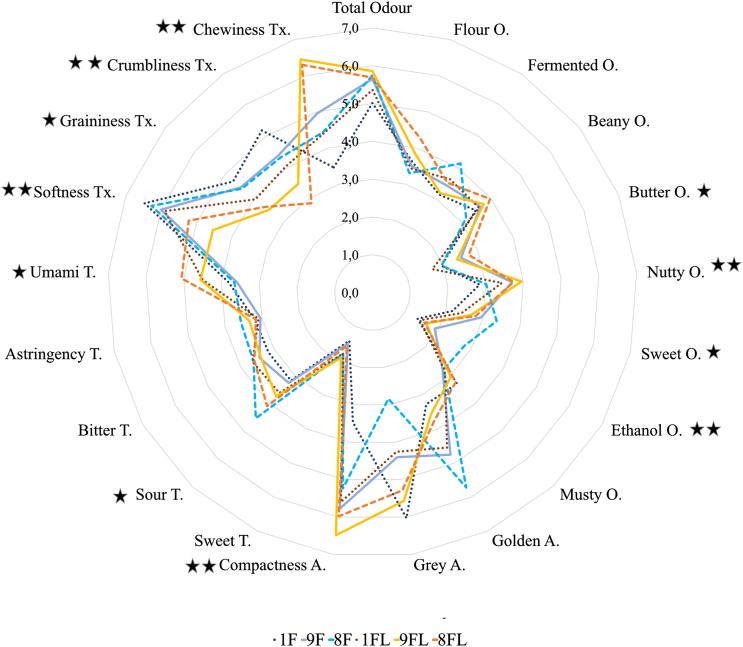
Sensory profile of tempeh-like products made from faba bean and a mixture of faba bean and whole-grain oat, pre-treated by soaking without or with *L. plantarum*, in cooked states. O: odour; A: appearance; T: taste; Tx: texture; presented as means of 3∗8 evaluations on a scale 0-10; statistically significance (*p < 0.05)* due to prototypes treatment and prototypes and LAB treatments.

The tempeh-like products made from faba bean and a mixture of faba bean and whole-grain oat in this study exhibited a diverse sensory profile, see Fig. 5. In general, the butter and flour odour attributes were relatively mild compared to the most intense notes of fermented, beany and sweet odours. However, nutty and ethanol odours were significantly different (*p < 0.05*) in both uncooked and cooked groups (Appendix A3 Table A3).

In terms of taste, the higher sourness compared to bitterness provided a better balance to the flavour, in tempeh-like products. Moreover, umami was the most intense taste perceived by the panel see Fig. 4. The texture of tempeh-like products was described as crumbly and grainy, with a noticeable chewiness and softness. The overall appearance of all the samples was compact, with significant differences in the colour as golden hue (due to the cooking/roasting process) with a slightly grey undertone.

In the cooked samples, LAB treatment resulted in a more intense overall odour, with a slight increase in beany and nutty notes. However, only nutty and ethanol odours showed a significant difference (*p < 0.05*) due to the LAB treatment in cooked sample (Appendix A3 Table A3). In terms of taste, sourness differed significantly across samples (p = 0.006), and umami intensity was significantly affected by soaking with LAB treatment (p = 0.001), reaching up to **5.1 in 8FL** compared to **3.8** in F8 (no LAB); the same tendency was observed in the other tempeh-like products (Fig. 4). However, no significant differences were found due to the interaction effect between prototype and LAB treatments. **Sweetness** and **sourness** were slightly higher in LAB group, while **bitterness** and **astringency** remained similar between both groups.

In general, all odour attributes were perceived as slightly more intense in the cooked samples compared to the uncooked ones. On the other hand, the **ethanol** odour decreased significantly (*p < 0.05*) in the LAB compared to No LAB, while the **fermented** odour showed a slight but non-significant reduction. LAB and cooked group exhibited the highest overall odour intensity (Fig. 4, Table A3).

Significant sensory profile differences (*p < 0.05*) in cooked samples were highly associated with the effect of prototype formulations. Higher whole-grain oat content prototypes, such as 85 % faba bean +15 % whole-grain oat (8F and 8FL), generally exhibit higher sourness, elevated nutty, sweet, butter, and ethanol odours, as well as increased chewiness, and compactness in texture (Fig. 4). In contrast, the 100 % faba bean formulation (1F) retains softer textures and milder flavour profiles. These differences suggested that the inclusion of whole-grain oat notably influenced the overall sensory profile, intensifying specific odours, tastes, and texture characteristics in cooked tempeh.

Regarding texture described by the panel, all attributes as graininess, crumbliness, softness, and chewiness were significantly different (*p < 0.05*) due to LAB treatment. For instance, 1F and 1FL had significantly lower chewiness (*p < 0.05*) values of 3.5 and 4.4 compared to 8F and 8FL with 4.4 and 6.3 (in average), respectively. However, the highest chewiness values of 6.5 and 6.3 were reported in samples 9FL and 8FL, respectively. These results are related to appearance analysis, where the compactness attribute was significantly higher (*p < 0.05*) in all samples with LAB compared to those without LAB. Compactness was defined by the panellists in the training session as the position of the beans close to each other by the mycelium. Higher compactness values may consider as a positive attribute that contributes to an appealing texture. Photographs of uncooked and cooked tempeh samples, both without and with LAB, are presented in supplementary material Appendix A4.

Descriptive analysis helps create a detailed sensory profile by evaluating key attributes such as appearance, texture, and flavour, while highlighting differences between them as observed in the spider chart in Fig. 4 and Table A3. However, training for this process is resource-intensive and may not always align with consumer perceptions (Tuccillo et al., 2022a).

#### 3.2.2 Affective test

The hedonic scale test was conducted using a nine-point balanced scale with 107 participants from Sweden and Finland. Only the cooked samples pre-treated with LAB during soaking were included as 1FL, 9FL and 8FL. This selection was based on their balanced sensory profiles, as identified by the semi-trained panel, thus, their potential for higher likability compared to the tempeh-like products without *L. plantarum* soaking. Moreover, the analysis was streamlined by reducing the number of samples from six to three, considering that the hedonic panel was not trained. This approach allowed for a detailed comparison of the effect of the raw materials used in the tempeh-like products on likability.

**Fig. 6:**
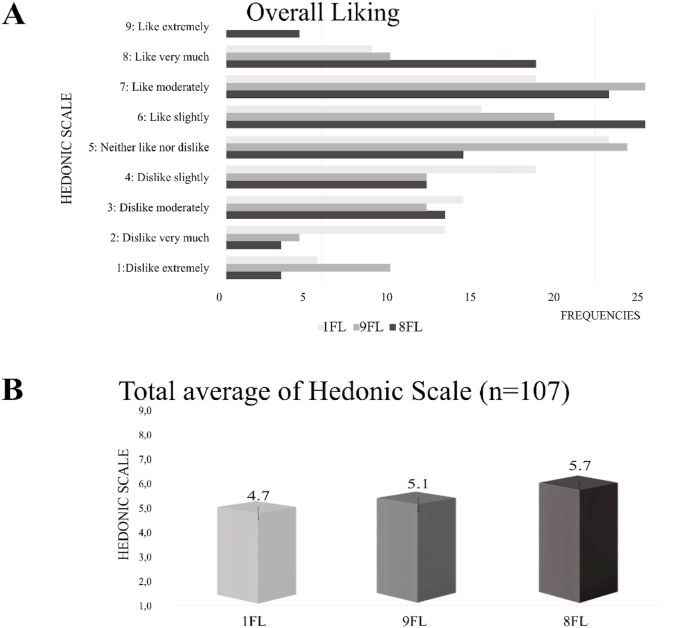
Affective test results on cooked tempeh samples, A. Distribution of overall liking scores and B. Total average hedonic scores of tempeh-like products made with *L. plantarum* soaking treatment, evaluating 1FL: 100 % faba bean, 9FL: 92 % faba bean 8 % whole-grain oats, and 8FL: 85 % faba bean 15 % whole-grain oats mixtures (n = 107).

The data is presented with frequency data table and overall average mean in Fig. 6; a significant difference was found among all three samples.

The addition of higher concentration of whole-grain oat to the faba bean base to produce tempeh positively impacted the acceptance scores, with 8FL (85 % faba bean + 15 % whole-grain oat) receiving the highest score of average 5.7 compared to only faba bean (4.7) and 92 % faba bean + 8 % whole-grain oat samples (5.1) (1FL and 9FL, respectively). This is a favourable outcome compared to the unprocessed faba bean flour or its fractions (Tuccillo et al., 2024), showing an enhancement of sensory qualities due to fermentation.

While analysing the sensory profile of the cooked 8FL sample with LAB, it was noted to have the lowest intensity in bitterness and sweetness, but a high intensity in sourness and the highest umami score among all samples. 8FL sample also received the second highest chewiness score from the panel, which aligns with its low crumbly and grainy texture rating.

Compared to soybean tempeh, which is the most commonly sold variety worldwide and in Sweden, some studies have reported to have an average acceptance score of 7.91 soybean tempeh deep fried (Rahmawati et al., 2021) and 5.5 soybean tempeh air fried with no oil addition (Dahlan et al., 2022); the scores for 1FL and 9FL tempeh in our study remained relatively lower. On the other hand, red kidney bean tempeh has been reported with an overall acceptance score of 4.21 and the combination of soybean with red kidney (75:25) shows a higher score of 7.18 in deep oil fried conditions (Rahmawati et al., 2021). In another study, velvet bean tempeh obtained a 3.04 score on the hedonic scale while soybean had 6.04 and the combination of soybean with velvet bean (70:30) reported a score of 5.48 (Fitri et al., 2024). As discussed previously, the cooking method is an important consideration in this study as no oil was added; alternative preparation methods could enhance the sensory profile. Thus, faba bean and whole-grain oat present a promising alternative for producing tempeh-like foods, reducing the dependence on soybean.

### 3.3 Physical properties: TPA and moisture content

The analyses for physical properties included moisture content and texture profiling are presented in Table 2. Moisture content ranged from 63.2 % to 69.9 % among all samples, where uncooked samples generally had a higher moisture than cooked ones, probably due to water evaporation during the heat processing. Compared to our previous study on faba bean tempeh, these faba bean and faba bean-whole-grain oat tempeh prototypes showed slightly higher moisture content in the uncooked group. However, in this study, a similar trend of lower moisture values was observed in samples with LAB treatment across both uncooked and cooked groups compared to our previous research (Fernandez Castaneda et al., 2024).

The highest chewiness from sensory evaluation by the semi-trained panel was observed in sample 9FL, with a corresponding high TPA chewiness (N), though this association was not consistent for samples 8F and 8FL across both methods. Nevertheless, both sensory and TPA analysis identified that faba bean and whole-grain oat mix samples had higher chewiness. Additionally, higher moisture content was associated with lower hardness and greater softness, consistent across TPA and sensory assessments.

Tempeh soaked with LAB showed notable texture improvements, with higher chewiness alongside reduced graininess and crumbliness compared to untreated samples. Enhanced mycelium formation by *R. microsporus* during SSF likely contributed to a more cohesive texture, making the product more appealing.

From another perspective, the multivariate analysis highlights associations between VOCs (uncooked and cooked) and sensory attributes (cooked), as shown in Appendix A5 (Figs. A2 and A3). The sensory PCA explains more variance (76.9 %) than the VOC PCA (51.3 %), indicating stronger differentiation in sensory data. The PLS analysis clarifies relationships between physicochemical properties (VOCs, texture, moisture) and sensory attributes, as shown in Appendix A5 (Fig. A4). For instance, umami taste and flour and beany odour are positively linked to certain esters and alcohol VOCs, while crumbliness and softness are negatively related and associated with 100 % faba bean tempeh NO LAB samples. These results suggest volatiles play a crucial role in shaping sensory profiles and could inform strategies for optimising sensory characteristics in tempeh-like products.

## 4 Conclusion

The study evaluated the effects of raw materials and soaking pre-treatment without and with *L. plantarum* on the volatile and sensory profiles of uncooked and cooked tempeh-like products. A total of 65 VOCs, including 3-methyl-1-butanol, ethanol, and acetic acid, were grouped into esters, alcohols, aldehydes, ketones, acids, pyrazines, and others. LAB pre-treatment reduced pungent alcohols (e.g., 3-methyl-1-butanol) and increased desirable compounds such as ketones, aldehydes, esters and acids contributing to fermented, nutty, buttery, sour, roasted, and umami notes.

LAB pre-treatment combined with SSF using *R. microsporus* enhanced odour complexity, masking beany and musty odours, while increasing umami taste and sourness (*p < 0.05*). Bitterness and astringency remained steady, but increasing umami taste and sourness modulated the off-flavours.

Significant texture changes were observed in LAB treated tempeh, as shown by sensory analysis and TPA. The inclusion of whole-grain oat and LAB-soaked treatments increased chewiness and reduced graininess, especially in samples with 85 % faba bean +15 % whole-grain oat treated with LAB. This tempeh-like product exhibited enhanced volatile, sensory, and texture profiles, leading to greater likability compared to only faba bean tempeh amongst the participants living in Sweden and Finland.

This study offers important insights into the role of different raw material combinations and fermentation processes, including microbial inoculation as a pre-treatment, in shaping the flavour profile of tempeh made from faba beans and faba bean–whole-grain oat mixtures. It highlights the potential for producing sustainable and high-quality tempeh-like products with an enhanced flavour.

## Funding

The authors acknowledge financial support from the project HealthFerm, which is co-funded by the 10.13039/501100000780European Union under the 10.13039/100018693Horizon Europe grant agreement No. 101060247 and from the Swiss 10.13039/501100007352State Secretariat for Education, Research and Innovation (SERI) under contract No. 22.00210. Views and opinions expressed are however those of the author(s) only and do not necessarily reflect those of the European Union nor European Research Executive Agency (REA). Neither the European Union nor REA can be held responsible for them. Moreover, this study was financially supported by Trees and Crops for the Future (TC4F), a Strategic Research Area at 10.13039/501100004360SLU, supported by the Swedish Government. 10.13039/100014294KH acknowledges funding from 10.13039/501100004012Jane and Aatos Erkko Foundation.

## CRediT authorship contribution statement

**Laura Alejandra Fernandez Castaneda:** Writing – original draft, preparation, Conceptualization, Methodology, Validation, Formal analysis, Investigation, Data curation, Writing – review & editing, Visualization. **Shania Saini:** Conceptualization, Methodology, Formal analysis, Investigation, Writing – review & editing. **Oskar Laaksonen:** Methodology, Resources, Writing – review & editing, Supervision. **Anna Kårlund:** Methodology, Resources. **Su-lin L. Leong:** Conceptualization, Resources, Writing – review & editing, Supervision. **William R. Newson:** Resources, Writing – review & editing, Supervision. **Volkmar Passoth:** Resources, Writing – review & editing, Supervision. **Kati Hanhineva:** Resources, Writing – review & editing, Funding acquisition. **Maud Langton:** Conceptualization, Methodology, Validation, Resources, Writing – review & editing, Supervision, Project administration, Funding acquisition. **Galia Zamaratskaia:** Conceptualization, Methodology, Validation, Resources, Data curation, Writing – review & editing, Visualization, Supervision, Funding acquisition, All authors have read and agreed to the published version of the manuscript.

## Declaration of competing interest

The authors declare that they have no known competing financial interests or personal relationships that could have appeared to influence the work reported in this article.

## Figures and Tables

**figs1:**
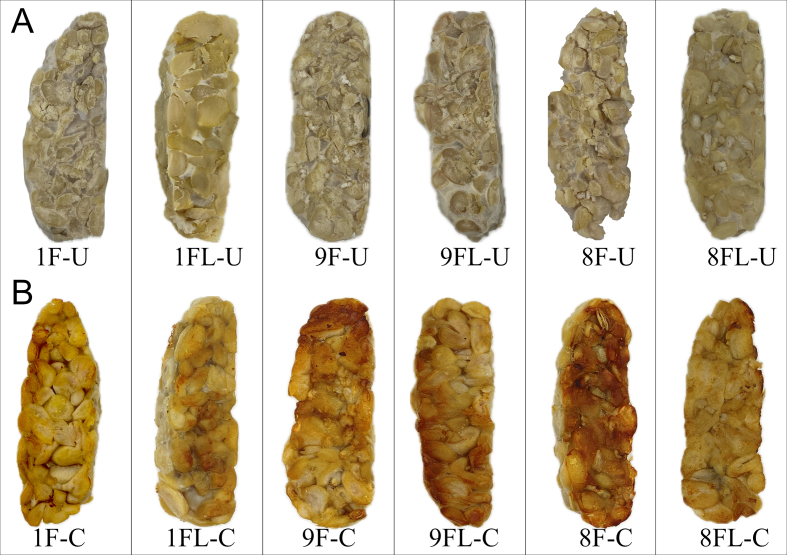
Figure S1 Picture of tempeh-like products corresponding to 1F = 100% faba bean, 9F = 92% faba bean + 8% whole-grain oat and 8F = 85% faba bean + 15% whole-grain oat without and with L = *L. plantarum*. –U = uncooked and –C = cooked. All cooked samples were roasted in a frying pan for 2 min per side.

**figs2:**
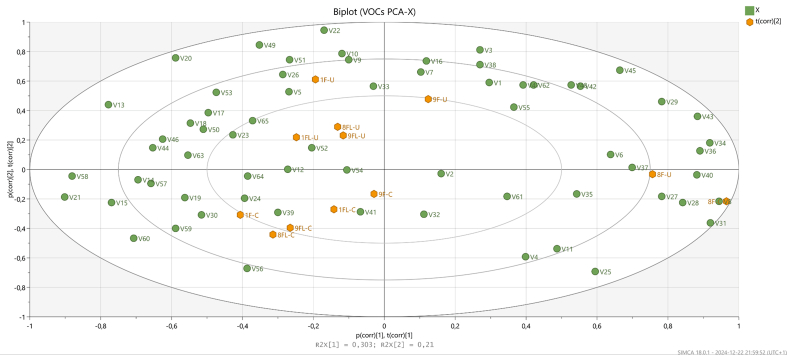
Figure S2 Biplot after principal component analysis (PCA) of volatiles organic compounds in tempeh-like samples. X-data font in green represent each VOC and the yellow font are samples 1F: 100% faba bean, 9F: 92% faba bean 8% whole-grain oats, and 8F: 85% faba bean 15% whole-grain oats mixtures, L: LAB, -U: uncooked, -C: cooked.

**figs3:**
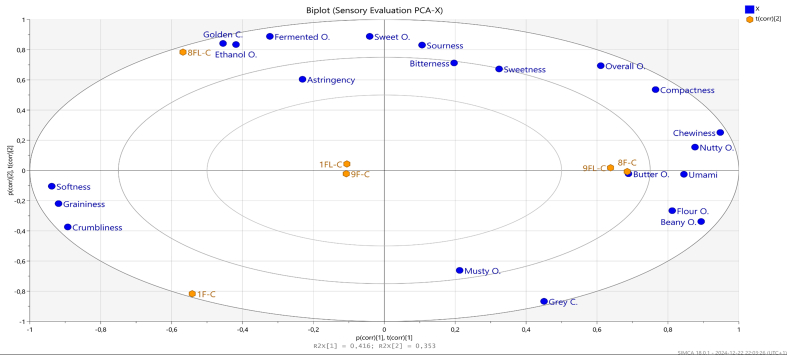
Figure A3 Biplot after principal component analysis (PCA) of sensory attribute in tempeh-like samples. X-data font in blue represent each attribute as O: odour and C: colour. The yellow font are samples 1F: 100% faba bean, 9F: 92% faba bean 8% whole-grain oats, and 8F: 85% faba bean 15% whole-grain oats mixtures, L: LAB, all -C: cooked since the sensory panel taste the samples.

**figs4:**
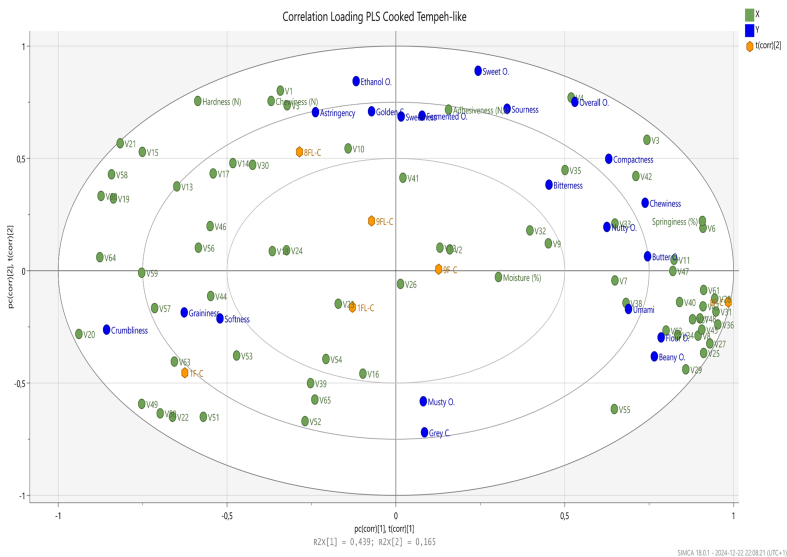
Figure A4 Correlation biplot of partial least squares (PLS) regression model on tempeh-like products cooked, yellow font are samples 1F: 100% faba bean, 9F: 92% faba bean 8% whole-grain oats, and 8F: 85% faba bean 15% whole-grain oats mixtures without and with L: *L. plantarum*. –C: cooked. Interaction between the physicochemical analysis (X-data green font; including volatiles organic compounds, TPA analysis and moisture content) and the sensory attributes (Y-data blue font, O: odour and C: colour)

## Data Availability

Data will be made available on request.
