# Supplementary material for: Sensory and volatile compound profiles in tempeh-like products from faba bean and oats
Source: Curr Res Food Sci. 2025 Mar 17;10:101029. doi: 10.1016/j.crfs.2025.101029 (PMC11987698; doi:10.1016/j.crfs.2025.101029)
Supplement: Multimedia component 1 [file mmc1.docx]

# Appendix A. Supplementary data

## A1 Panel training and standard references samples

Table A1 Overview of sensory attributes source of references, description and intensity scale

| **Descriptor category** | **Perceived**  **attribute** | **Source of reference** | **Description** | **Intensity scale** |
| --- | --- | --- | --- | --- |
| Odour | Overall odour | Commercial faba bean tempeh | Intensity of the whole odour experience | n.a. |
|  | Flour/grain | 4 g Lupin flour | Milled dried pulses | n.a. |
|  | Fermented | 12.5% Dried baker yeast in water | Yeast, tangy, sour | n.a. |
|  | Beany | 2g Chickpeas (canned) | Green pea, boiled pulses | n.a. |
|  | Butter | 2g Butter | Creamy, dairy and fatty | n.a. |
|  | Nutty | 4g Crushed hazelnuts | Slightly sweet and toasted | n.a. |
|  | Sweet | 2g Crushed caramelised | Sweet and toasted undertones | n.a. |
|  | Ethanol | 50 µL of 70% Ethanol on 1*1cm^2^ filter paper | Mild alcohol solution, pungent and fruity undertones | n.a. |
|  | Musty | 25% Faba bean flour in water | Stale, stuffy, not fresh | n.a. |
| Taste | Sweet | 1% of sucrose |  | 3 |
|  | Sour | 0.06% Citric acid |  | 5 |
|  | Bitter | 0.03% Caffeine |  | 4 |
|  | Astringent | 0.03% Aluminium sulphate |  | 4 |
|  | Umami | 0.06% Monosodium glutamate |  | 5 |
| Texture | Softness | Mini marshmallow (4 pieces) | Easy of fracture-ability | n.a. |
|  | Graininess | 81% Rye bread (pieces) | Rough and course | n.a. |
|  | Crumbly | Oat biscuit | Breaking into little pieces | n.a. |
|  | Chewiness | Liquorice candy (Fazer) | Bounce, elasticity, resistance while chewing | n.a. |
| Appearance | Golden | n.a. | Yellow colour | n.a. |
|  | Grey | n.a. | Dark colour | n.a. |
|  | Compactness | n.a. | Position of the beans close to each other by the mycelium | n.a. |
| n.a.= not applicable  The intensity scale was from 0 as “absence” and 10 as “maximum intensity”. | | | | |
|  | | | | |

## A2 Volatile organic compounds

Table A2 Volatile organic compounds semi-quantified by HS-SPME-GC-MS in tempeh prototypes made from faba bean, and mix of faba bean and whole-grain oat pre-treated with *L. plantarum*. Uncooked and Cooked groups.

| TREATMENT= UNCOOKED | | | | | | | | | |
| --- | --- | --- | --- | --- | --- | --- | --- | --- | --- |
| Prototype | **1F** | | **9F** | | **8F** | | ***p-value*, Prototype** | ***p-value*, LAB** | ***p-value*, Prototype* LAB** |
|  | **100% Faba bean** | | **92%Faba bean+**  **8%whole-grain oat** | | **85%Faba bean+**  **15%whole-grain oat** | |  |  |  |
| Soaking | **No LAB** | **LAB** | **No LAB** | **LAB** | **No LAB** | **LAB** |  |  |  |
| *Aldehydes (12)* | | |  |  |  |  |  |  |  |
| V1 | 13.06 (10.2-16.73) | 13.87 (10.83-17.76) | 25.89 (20.22-33.16) | 14.48 (11.31-18.55) | 23.53 (18.37-30.14) | 12.13 (9.47-15.53) | 0.023 | 0.001 | **0.015** |
| V2 | 1.04 (0.32-3.31) | 0.21 (0.07-0.68) | 1.91 (0.6-6.09) | 0.94 (0.3-3.01) | 1.24 (0.39-3.97) | 0.95 (0.3-3.04) | 0.157 | 0.074 | 0.474 |
| V3 | 11 (6.89-17.54) | 9.75 (6.11-15.56) | 27.98 (17.54-44.64) | 15.66 (9.82-24.98) | 11.55 (7.24-18.43) | 10.8 (6.77-17.23) | 0.012 | 0.169 | 0.445 |
| V4 | 0.57 (0.39-0.81) | 0.29 (0.2-0.41) | 0.81 (0.56-1.17) | 0.21 (0.15-0.31) | 2.02 (1.4-2.91) | 0.73 (0.5-1.05) | <0.001 | <0.001 | 0.188 |
| V5 | 2.35 (0.26-20.85) | 1.33 (0.15-11.77) | 3.49 (0.39-31.01) | 1.59 (0.18-14.13) | 1.07 (0.12-9.47) | 0.26 (0.03-2.35) | 0.323 | 0.285 | 0.914 |
| V6 | 1.15 (0.78-1.69) | 0.6 (0.41-0.89) | 2.05 (1.4-3.01) | 0.72 (0.49-1.06) | 1.32 (0.9-1.94) | 1.64 (1.12-2.41) | 0.021 | 0.005 | **0.011** |
| V7 | 2.13 (0.64-7.13) | 1.14 (0.34-3.83) | 4.24 (1.26-14.2) | 1.41 (0.42-4.71) | 0.1 (0.03-0.32) | 1.61 (0.48-5.41) | 0.017 | 0.435 | **0.008** |
| V8 | 0.13 (0-139.42) | 0 (0-0.08) | 0.33 (0-367.76) | 0.2 (0-221.04) | 1.74 (0-1929.15) | 0.16 (0-175.16) | 0.257 | 0.211 | 0.548 |
| V9 | 1.66 (0.66-4.18) | 1.04 (0.41-2.61) | 0.67 (0.26-1.68) | 0.63 (0.25-1.59) | 0.7 (0.28-1.75) | 0.6 (0.24-1.5) | 0.197 | 0.525 | 0.875 |
| V10 | 4.98 (3.2-7.77) | 2.2 (1.41-3.44) | 6.58 (4.22-10.25) | 2.88 (1.84-4.48) | 0.96 (0.61-1.49) | 1.3 (0.83-2.03) | <0.001 | 0.020 | **0.025** |
| V11 | 0.21 (0-266.42) | 0.31 (0-394.64) | 0.51 (0-646.58) | n.d. | 0.9 (0-1146.55) | n.d. | 0.014 | 0.000 | **0.007** |
| *Ketones (11)* | | | |  |  |  |  |  |  |
| V12 | 7.74 (1.41-42.55) | 7.79 (1.42-42.84) | 8.04 (1.46-44.22) | 6.51 (1.19-35.81) | 1.58 (0.29-8.7) | 0.6 (0.11-3.33) | 0.034 | 0.554 | 0.813 |
| V13 | 28.52 (22.96-35.43) | 26.21 (21.1-32.56) | 18.56 (14.94-23.06) | 29.44 (23.7-36.58) | 3.9 (3.14-4.85) | 26.61 (21.42-33.06) | <0.001 | <0.001 | **<0.001** |
| V14 | 52.57 (47.01-58.78) | 84.96 (75.98-94.99) | 81.77 (73.13-91.43) | 98.93 (88.48-110.62) | 16.23 (14.52-18.15) | 55.21 (49.38-61.73) | <0.001 | <0.001 | **<0.001** |
| V15 | 0.28 (0-159.25) | 0.27 (0-155.02) | 0.1 (0-57.02) | 0.42 (0-238.1) | n.d. | 0.11 (0-0.08) | 0.000 | 0.050 | 0.061 |
| V16 | 13.84 (9.76-19.63) | 9.85 (6.94-13.97) | 8.14 (5.74-11.55) | 9.41 (6.63-13.35) | 12.84 (9.05-18.21) | 14.3 (10.08-20.29) | 0.052 | 0.828 | 0.280 |
| V17 | 0.77 (0-301.59) | 0.75 (0-292.8) | 0 (0-0.02) | 0.39 (0-154.51) | n.d. | 0.77 (0-300.28) | 0.005 | 0.001 | **0.004** |
| V18 | 11.27 (6.91-18.37) | 11.09 (6.8-18.08) | 9.04 (5.55-14.74) | 9.81 (6.02-16) | 1.48 (0.91-2.41) | 17.98 (11.03-29.32) | 0.012 | 0.001 | **0.000** |
| V19 | 0.43 (0.2-0.91) | 0.48 (0.23-1.01) | 0.19 (0.09-0.4) | 0.39 (0.19-0.83) | 0.31 (0.15-0.66) | 0.49 (0.23-1.04) | 0.355 | 0.154 | 0.680 |
| V20 | 1.43 (1-2.05) | 1.12 (0.78-1.6) | 1.28 (0.9-1.83) | 0.97 (0.67-1.38) | 0.49 (0.35-0.71) | 1.28 (0.89-1.82) | 0.040 | 0.326 | **0.004** |
| V21 | 0.19 (0.15-0.25) | 0.24 (0.18-0.3) | 0.16 (0.13-0.21) | 0.3 (0.24-0.39) | n.d. | 0.26 (0.2-0.33) | <0.001 | <0.001 | **<0.001** |
| V22 | 63.13 (51.71-77.09) | 50.02 (40.96-61.07) | 67.72 (55.46-82.69) | 42.93 (35.16-52.42) | 31.09 (25.46-37.96) | 48.49 (39.72-59.21) | 0.003 | 0.298 | **0.001** |
| V23 | 0.49 (0.31-0.78) | 0.36 (0.23-0.57) | 0.42 (0.26-0.66) | 0.31 (0.19-0.48) | 0.11 (0.07-0.17) | 0.21 (0.14-0.34) | 0.001 | 0.882 | 0.055 |
| *Ester (15)* | |  |  |  |  |  |  |  |  |
| V24 | 1.39 (1.09-1.79) | 1.68 (1.31-2.16) | 1.96 (1.53-2.51) | 1.53 (1.19-1.96) | 1.23 (0.96-1.58) | 1.32 (1.03-1.69) | 0.061 | 0.972 | 0.185 |
| V25 | 2.66 (1.78-3.98) | 3.08 (2.06-4.6) | 3.81 (2.55-5.68) | 2.81 (1.88-4.2) | 12.33 (8.26-18.42) | 4.27 (2.86-6.38) | 0.001 | 0.019 | **0.020** |
| V26 | 8.56 (6.37-11.51) | 9.21 (6.85-12.38) | 11.33 (8.43-15.24) | 8.38 (6.23-11.27) | 5.05 (3.75-6.79) | 11.12 (8.27-14.96) | 0.188 | 0.118 | **0.005** |
| V27 | 0.09 (0-614.23) | 0 (0-0.91) | 0.11 (0-693.6) | 0.05 (0-357.32) | 0.25 (0-1632.1) | 0.03 (0-157.32). | 0.338 | 0.046 | 0.251 |
| V28 | 4.85 (1.79-13.11) | 4.98 (1.84-13.46) | 4.05 (1.5-10.96) | 3.3 (1.22-8.92) | 5.26 (1.94-14.24) | 3.2 (1.18-8.67) | 0.812 | 0.558 | 0.850 |
| V29 | 0.21 (0.06-0.73) | 0.22 (0.06-0.76) | 0.37 (0.11-1.26) | 0.18 (0.05-0.61) | 0.37 (0.11-1.28) | 0.21 (0.06-0.72) | 0.899 | 0.386 | 0.782 |
| V30 | 0.73 (0.53-1) | 0.43 (0.31-0.59) | 1.37 (1-1.88) | 1.54 (1.12-2.11) | 0.08 (0.06-0.11) | 1.66 (1.21-2.28) | <0.001 | <0.001 | **<0.001** |
| V31 | 3.34 (2.68-4.17) | 3.73 (3-4.65) | 5.02 (4.03-6.26) | 5.56 (4.47-6.93) | 16.16 (12.97-20.13) | 6.25 (5.02-7.79) | <0.001 | 0.012 | **0.000** |
| V32 | 0.67 (0.48-0.92) | 0.84 (0.61-1.16) | 0.61 (0.44-0.84) | 1.22 (0.89-1.68) | 0.7 (0.51-0.96) | 1.25 (0.91-1.72) | 0.340 | 0.001 | 0.291 |
| V33 | 4.54 (2.6-7.92) | 5.52 (3.16-9.64) | 8.66 (4.96-15.13) | 9.4 (5.39-16.42) | 3.88 (2.22-6.78) | 16.34 (9.36-28.53) | 0.091 | 0.018 | **0.039** |
| V34 | 1 (0.59-1.7) | 0.45 (0.27-0.77) | 1.32 (0.78-2.23) | 0.48 (0.28-0.82) | 7.65 (4.51-12.95) | 0.52 (0.31-0.88) | 0.002 | <0.001 | **0.004** |
| V35 | 10.88 (8.23-14.37) | 18.15 (13.74-23.97) | 7.28 (5.51-9.61) | 16.17 (12.24-21.36) | 29.35 (22.22-38.77) | 16.82 (12.73-22.21) | 0.000 | 0.033 | **0.001** |
| V36 | 0.54 (0.31-0.92) | 0.4 (0.23-0.69) | 0.52 (0.3-0.9) | 0.29 (0.17-0.5) | 0.77 (0.45-1.32) | 0.32 (0.19-0.55) | 0.632 | 0.015 | 0.531 |
| V37 | 1.47 (0.86-2.51) | 1.09 (0.64-1.87) | 0.59 (0.34-1) | 1.31 (0.77-2.24) | 1.49 (0.87-2.54) | 0.87 (0.51-1.49) | 0.338 | 0.966 | **0.040** |
| V38 | 2.7 (0.98-7.46) | 1.06 (0.38-2.93) | 2.02 (0.73-5.56) | 2.14 (0.78-5.91) | 1.75 (0.63-4.83) | 2.57 (0.93-7.08) | 0.869 | 0.673 | 0.368 |
| *Alcohol (13)* | |  |  |  |  |  |  |  |  |
| V39 | 2.62 (0-3864.24) | 4.26 (0-6273.04) | 4.05 (0-5962.99) | 7 (0-10318.7) | 2.86 (0-4217.13) | 3.21 (0-4.95) | 0.373 | 0.434 | 0.397 |
| V40 | 141.35 (123.95-161.19) | 157.11 (137.77-179.16) | 172.18 (150.99-196.36) | 192.59 (168.88-219.62) | 314.37 (275.67-358.5) | 230.44 (202.08-262.79) | <0.001 | 0.541 | **0.006** |
| V41 | 12.01 (10.8-13.36) | 22.59 (20.31-25.12) | 39.73 (35.72-44.18) | 28.78 (25.88-32.01) | 24.13 (21.7-26.84) | 18.96 (17.05-21.09) | <0.001 | 0.581 | **<0.001** |
| V42 | 2.12 (1.72-2.6) | 1.71 (1.39-2.1) | 3.79 (3.09-4.66) | 2.37 (1.93-2.91) | 3.7 (3.01-4.54) | 2.67 (2.17-3.28) | 0.000 | 0.001 | 0.419 |
| V43 | 46.04 (38.12-55.61) | 42.6 (35.27-51.45) | 57 (47.19-68.85) | 48.31 (40-58.35) | 85.68 (70.94-103.49) | 46.53 (38.52-56.2) | 0.005 | 0.002 | **0.021** |
| V44 | 42.69 (34.73-52.48) | 47.98 (39.03-58.98) | 39.09 (31.8-48.06) | 49.96 (40.64-61.42) | 27.76 (22.58-34.12) | 42.78 (34.8-52.59) | 0.025 | 0.005 | 0.283 |
| V45 | 521 (450.6-602.4) | 448.92 (388.26-519.05) | 468.62 (405.29-541.83) | 480.24 (415.35-555.27) | 551.23 (476.74-637.35) | 424.96 (367.53-491.35) | 0.944 | 0.036 | 0.141 |
| V46 | 2.91 (2.23-3.8) | 3.38 (2.59-4.4) | 1.04 (0.8-1.35) | 2.04 (1.57-2.67) | 0.99 (0.76-1.29) | 2.74 (2.1-3.57) | <0.001 | <0.001 | **0.012** |
| V47 | 1 (0.03-34.03) | 1.07 (0.03-36.51) | 3.19 (0.09-108.68) | 5.19 (0.15-176.85) | 22.54 (0.66-768.7) | 8.74 (6.02-25.13) | 0.632 | 0.484 | 0.441 |
| V48 | 25.55 (20.32-32.13) | 21.47 (17.07-27) | 54.5 (43.34-68.54) | 37.71 (29.99-47.42) | 27.96 (22.23-35.16) | 15.21 (12.1-19.13) | <0.001 | 0.001 | 0.160 |
| V49 | 3.45 (2.16-5.5) | 2.39 (1.5-3.81) | 1.16 (0.72-1.85) | 1.55 (0.97-2.47) | n.d. | 1.67 (1.05-2.67) | <0.001 | <0.001 | **<0.001** |
| V50 | 0.29 (0-2275.06) | 0 (0-1.24) | n.d. | n.d. | n.d. | 0.13 (0-1.19) | 0.003 | 0.518 | 0.060 |
| V51 | 0.61 (0-59664.35) | 0.71 (0-69251.92) | 0.08 (0-46.78) | 0.49 (0-47590.27) | 0.18 (0-19.06) | 0.16 (0-0.02) | 0.120 | 0.998 | 0.441 |
| *Acids (4)* |  |  |  |  |  |  |  |  |  |
| V52 | 0.46 (0-15812.06) | 0.54 (0-18494.56) | 0.06 (0-3.31) | 0.01(0-2.43) | 0.28 (0-9397) | 0 (0-3.15) | 0.071 | 0.219 | 0.649 |
| V53 | 22.6 (15.64-32.65) | 22.96 (15.89-33.18) | 10.83 (7.49-15.64) | 10.78 (7.46-15.58) | 4.6 (3.18-6.65) | 11.42 (7.9-16.5) | <0.001 | 0.046 | **0.030** |
| V54 | 0.02 (0.01-0.08) | 1.1 (0.29-4.13) | 0.9 (0.24-3.39) | 0.6 (0.16-2.25) | 0.38 (0.1-1.42) | 0.06 (0.02-0.24) | 0.037 | 0.258 | **0.001** |
| V55 | 6.4 (3.45-11.89) | 8.27 (4.46-15.35) | 9.39 (5.06-17.43) | 4.18 (2.25-7.77) | 3.51 (1.89-6.51) | 3.92 (2.11-7.28) | 0.082 | 0.538 | 0.169 |
| *Pyrazines (4)* | |  |  |  |  |  |  |  |  |
| V56 | n.d. | 0.58 (0-5.08) | n.d. | n.d. | n.d. | n.d. | 0.256 | 0.055 | 0.256 |
| V57 | 0.54 (0-229.99) | 0.3 (0-127.04) | 0.3 (0-127.99) | 0.14 (0-59.97) | 0.04 (0-0.03) | 0.55 (0-234.58) | 0.311 | 0.286 | 0.178 |
| V58 | 1.66 (1.02-2.68) | 1.2 (0.74-1.95) | 1.09 (0.68-1.77) | 1.49 (0.92-2.41) | 0.08 (0.05-0.14) | 1.71 (1.06-2.77) | <0.001 | 0.000 | **<0.001** |
| V59 | 0.55 (0.3-1.02) | 0.26 (0.14-0.49) | 0.39 (0.21-0.72) | 0.33 (0.18-0.6) | n.d. | 1.01 (0.55-1.88) | <0.001 | <0.001 | **<0.001** |
| *Others (6)* |  |  |  |  |  |  |  |  |  |
| V60 | 0.15 (0.08-0.3) | 0.43 (0.22-0.85) | 0.48 (0.24-0.94) | 0.3 (0.15-0.6) | 0.18 (0.09-0.35) | 0.4 (0.2-0.78) | 0.400 | 0.094 | 0.070 |
| V61 | 0.12 (0.09-0.16) | 0.19 (0.14-0.26) | 0.21 (0.15-0.28) | 0.24 (0.18-0.33) | n.d. | 0.12 (0.08-0.16) | <0.001 | <0.001 | **<0.001** |
| V62 | 3.51 (0-2485.73) | n.d. | 1.71 (0-1211.36) | 0.47 (0-330.08) | 1.8 (0-1275.44) | 0.91 (0-644.68) | 0.049 | 0.024 | **0.031** |
| V63 | 3.14 (0.01-1435.78) | 1.89 (0-862.45) | n.d. | 0.22 (0-100.73) | n.d. | 1.12 (0-511.04) | 0.002 | 0.000 | **0.004** |
| V64 | 3.58 (2.89-4.45) | 3.06 (2.46-3.8) | 1.08 (0.87-1.33) | 2.11 (1.7-2.62) | 2.97 (2.39-3.68) | 1.19 (0.96-1.48) | <0.001 | 0.128 | **<0.001** |
| *V65 | 1.49 | 0.25 | 0.59 | 0.79 | 0.12 | 0.20 | 0.2390 | 0.354 | 0.187 |
| TREATMENT= COOKED | | | | | | | | | |
| Prototype | **1F** | | **9F** | | **8F** | | *p-value*, Prototype | *p-value*, LAB | ***p-value*, Prototype*LAB** |
|  | **100% Faba bean** | | **92%Faba bean+**  **8%whole-grain oat** | | **85%Faba bean+**  **15%whole-grain oat** | |  |  |  |
| Soaking | **No LAB** | **LAB** | **No LAB** | **LAB** | **No LAB** | **LAB** |  |  |  |
| *Aldehydes (12)* | |  |  |  |  |  |  |  |  |
| V1 | 7.2 (5.91-8.76) | 9.07 (7.45-11.04) | 8.75 (7.19-10.65) | 9.65 (7.92-11.74) | 7.24 (5.94-8.81) | 9.81 (8.05-11.94) | 0.379 | 0.014 | 0.529 |
| V2 | 0.79 (0.46-1.35) | 1.33 (0.78-2.28) | 2.4 (1.4-4.11) | 1.3 (0.76-2.23) | 1 (0.59-1.71) | 0.97 (0.56-1.66) | 0.063 | 0.836 | 0.113 |
| V3 | 2.62 (1.8-3.83) | 3.84 (2.63-5.61) | 6.31 (4.32-9.21) | 4.82 (3.3-7.03) | 7.02 (4.81-10.25) | 5.87 (4.02-8.57) | 0.004 | 0.877 | 0.170 |
| V4 | 0.52 (0.34-0.78) | 0.75 (0.5-1.13) | 3.02 (2.01-4.53) | 1.94 (1.29-2.92) | 3.97 (2.64-5.96) | 5.94 (3.95-8.93) | <0.001 | 0.481 | 0.074 |
| V5 | 1.18 (0.87-1.61) | 0.63 (0.46-0.86) | 1.95 (1.43-2.65) | 1.39 (1.02-1.9) | 0.78 (0.57-1.06) | 2.56 (1.88-3.49) | 0.002 | 0.512 | **<0.001** |
| V6 | 0.76 (0.59-0.99) | 0.8 (0.62-1.04) | 1.21 (0.94-1.56) | 0.5 (0.38-0.64) | 2.26 (1.75-2.91) | 0.65 (0.51-0.84) | 0.003 | <0.001 | **0.000** |
| V7 | 0.52 (0.33-0.82) | 0.32 (0.2-0.51) | 2.38 (1.5-3.79) | 0.28 (0.17-0.44) | 1.88 (1.18-2.99) | 0.42 (0.27-0.67) | 0.006 | <0.001 | **0.007** |
| V8 | 0.2 (0.11-0.38) | 0.16 (0.09-0.31) | 0.24 (0.13-0.46) | 0.8 (0.42-1.51) | 2 (1.06-3.79) | 0.1 (0.05-0.19) | 0.014 | 0.015 | **<0.001** |
| V9 | 0.32 (0.13-0.78) | 0.51 (0.21-1.26) | 0.89 (0.36-2.2) | 2.6 (1.05-6.4) | 0.57 (0.23-1.4) | 1.83 (0.74-4.51) | 0.021 | 0.020 | 0.675 |
| V10 | 0.96 (0.6-1.55) | 1.09 (0.68-1.77) | 1.5 (0.93-2.41) | 0.5 (0.31-0.81) | 1.02 (0.63-1.65) | 0.27 (0.17-0.43) | 0.024 | 0.001 | **0.013** |
| V11 | 0.59 (0.37-0.94) | 0.34 (0.21-0.54) | 0.94 (0.59-1.51) | 0.25 (0.15-0.39) | 2.72 (1.69-4.37) | 0.15 (0.09-0.24) | 0.248 | <0.001 | **0.001** |
| *Ketones (11)* |  |  |  |  |  |  |  |  |  |
| V12 | 6.17 (4.7-8.1) | 5.42 (4.12-7.12) | 13.27 (10.1-17.43) | 7.07 (5.38-9.29) | 6.36 (4.84-8.35) | 6.52 (4.97-8.57) | 0.003 | 0.034 | 0.055 |
| V13 | 17 (14.02-20.6) | 13.86 (11.44-16.8) | 21.06 (17.37-25.52) | 25.73 (21.23-31.19) | 3.9 (3.22-4.73) | 18.24 (15.05-22.1) | <0.001 | <0.001 | **<0.001** |
| V14 | 59.66 (24.95-142.61) | 31.33 (13.1-74.89) | 95.2 (39.82-227.59) | 127.6 (53.37-305.03) | 13.68 (5.72-32.7) | 77.47 (32.41-185.21) | 0.025 | 0.184 | **0.035** |
| V15 | 0.32 (0.22-0.46) | 0.23 (0.16-0.33) | 0.36 (0.25-0.52) | 0.48 (0.33-0.69) | 0.45 (0.31-0.65) | n.d. | <0.001 | <0.001 | **<0.001** |
| V16 | 8.6 (6.94-10.67) | 4.54 (3.66-5.63) | 5.8 (4.68-7.19) | 4.74 (3.82-5.87) | 7.2 (5.81-8.92) | 6.5 (5.24-8.06) | 0.053 | 0.002 | **0.042** |
| V17 | 0.34 (0.26-0.44) | 0.31 (0.23-0.4) | 0.29 (0.22-0.38) | 0.31 (0.24-0.4) | 0.28 (0.22-0.37) | 0.23 (0.18-0.31) | 0.226 | 0.466 | 0.599 |
| V18 | 10.66 (6.79-16.74) | 4.02 (2.56-6.32) | 20.13 (12.82-31.6) | 4.61 (2.94-7.23) | 11.06 (7.04-17.36) | 1.77 (1.13-2.78) | 0.009 | 0.247 | **<0.001** |
| V19 | 0.52 (0.34-0.79) | 0.49 (0.32-0.75) | 0.14 (0.09-0.21) | 0.43 (0.091-1.48) | 0.69 (0-1.23) | 0.06 (0.04-0.09) | 0.008 | <0.001 | **<0.001** |
| V20 | 1.01 (0-648.02) | 0.76 (0-483.98) | 0.51 (0-329.68) | 0.67 (0-1.61) | 0.57 (0-251.86) | 0.09 (0-1.61) | 0.031 | 0.077 | **0.037** |
| V21 | 0.26 (0-84.42) | 0.22 (0-72.54) | 0.23 (0-75.13) | 0.19 (0-63.64) | 0.39 (0-1.61) | 0.01 (0-1.61) | 0.021 | 0.046 | **0.022** |
| V22 | 28.4 (0.06-13746.85) | 17.54 (0.04-8490.59) | 18.17 (0.04-8795.9) | 14.08(5.67-23.45) | 13.32 (0.03-6445.23) | 15.74(4.67-53.45) | 0.002 | <0.001 | **0.002** |
| V23 | 0.36 (0.21-0.62) | 0.31 (0.18-0.53) | 1.22 (0.71-2.11) | 0.08 (0.05-0.14) | 0.19 (0.11-0.33) | 0.36 (0.21-0.61) | 0.613 | 0.003 | **<0.001** |
| *Esters (15)* |  |  |  |  |  |  |  |  |  |
| V24 | 1.24 (0.88-1.76) | 3.03 (2.14-4.3) | 1.96 (1.38-2.78) | 1.94 (1.37-2.76) | 1.74 (0.66-2.33) | 0.97 (1.21-2.44) | 0.032 | 0.003 | **0.044** |
| V25 | 7.98 (5.18-12.3) | 17.73 (11.5-27.33) | 14.23 (9.23-21.93) | 11.25 (7.3-17.34) | 6.29 (4.08-9.69) | 49.26 (31.96-75.93) | 0.148 | 0.010 | **<0.001** |
| V26 | 5.97 (4.57-7.8) | 7.59 (5.81-9.92) | 10.24 (7.83-13.38) | 7.02 (5.37-9.18) | 5.55 (4.24-7.25) | 5.41 (4.14-7.07) | 0.013 | 0.601 | 0.078 |
| V27 | 0.07 (0.04-0.12) | 0.15 (0.09-0.26) | 0.34 (0.2-0.6) | 0.1 (0.06-0.18) | 0.04 (0.02-0.07) | 2.32 (1.34-4.02) | 0.003 | <0.001 | **<0.001** |
| V28 | 2.24 (0.61-8.25) | 6.08 (1.65-22.41) | 2.66 (0.72-9.83) | 3.62 (0.98-13.34) | 5.28 (1.43-19.49) | 76.97 (20.86-283.95) | 0.016 | 0.368 | **0.022** |
| V29 | 0.05 (0.03-0.11) | 0.15 (0.07-0.29) | 0.21 (0.1-0.42) | 14.06 (6.96-28.38) | 1.85 (0.92-3.74) | 15.72 (7.79-31.75) | <0.001 | <0.001 | **0.001** |
| V30 | 1.73 (1.09-2.76) | 0.45 (0.29-0.72) | 2.9 (1.82-4.62) | 0.31 (0.2-0.5) | 0.13 (0.08-0.21) | 1.13 (0.71-1.79) | 0.002 | 0.020 | **<0.001** |
| V31 | 5.46 (4.69-6.35) | 7.95 (6.83-9.25) | 7.81 (6.71-9.09) | 6.83 (5.87-7.96) | 18.92 (16.25-22.03) | 6.32 (5.43-7.35) | <0.001 | 0.000 | **<0.001** |
| V32 | 0.85 (0.62-1.17) | 1.14 (0.83-1.56) | 0.51 (0.37-0.71) | 16.34 (11.89-22.43) | 1.4 (1.02-1.92) | 6.24 (4.54-8.57) | <0.001 | <0.001 | **<0.001** |
| V33 | 2.4 (1.21-4.75) | 3.81 (1.92-7.54) | 2.33 (1.18-4.62) | 0.6 (0.31-1.2) | 5.04 (2.55-9.99) | 0.88 (0.45-1.75) | 0.035 | 0.005 | **0.010** |
| V34 | 0.25 (0.15-0.42) | 0.61 (0.36-1.03) | 1.41 (0.83-2.38) | 2.39 (1.42-4.05) | 9.08 (5.37-15.35) | 2.31 (1.36-3.89) | <0.001 | 0.933 | **0.001** |
| V35 | 9.35 (8.52-10.26) | 14.57 (13.27-15.99) | 8.01 (7.3-8.79) | 14.48 (13.19-15.9) | 18.14 (16.52-19.91) | 19.4 (17.67-21.29) | <0.001 | <0.001 | **<0.001** |
| V36 | 0.26 (0.15-0.46) | 0.31 (0.18-0.56) | 0.49 (0.28-0.87) | 36.23 (20.37-64.43) | 42.22 (23.74-75.08) | 2.49 (1.4-4.43) | <0.001 | <0.001 | **<0.001** |
| V37 | 0.94 (0.46-1.93) | 1.07 (0.52-2.21) | 0.58 (0.28-1.18) | 0.89 (0.43-1.83) | 0.96 (0.47-1.97) | 3.19 (1.55-6.56) | 0.056 | 0.447 | 0.064 |
| V38 | 0.64 (0.48-0.85) | 0.76 (0.58-1.02) | 2.89 (2.18-3.84) | 0.91 (0.69-1.21) | 0.54 (0.41-0.72) | 1.82 (1.37-2.41) | 0.000 | <0.001 | **0.000** |
| *Alcohol (13)* | |  |  |  |  |  |  |  |  |
| V39 | 5.33 (3.61-7.85) | 7.21 (4.89-10.63) | 5.83 (3.96-8.6) | 5.7 (3.87-8.4) | 2.69 (1.82-3.96) | 3.19 (2.16-4.7) | 0.002 | 0.805 | 0.424 |
| V40 | 153.57 (141.28-166.94) | 164.39 (151.23-178.7) | 157.9 (145.26-171.64) | 130.63 (120.17-142) | 178.06 (163.8-193.56) | 300.49 (276.43-326.64) | <0.001 | <0.001 | **<0.001** |
| V41 | 18.65 (17.15-20.29) | 27.13 (24.95-29.52) | 48.45 (44.54-52.7) | 44.63 (41.03-48.55) | 27.31 (25.1-29.7) | 21.04 (19.34-22.88) | <0.001 | <0.001 | **0.000** |
| V42 | 0.98 (0.82-1.16) | 1.01 (0.85-1.2) | 1.26 (1.06-1.5) | 1.27 (1.07-1.51) | 1.93 (1.62-2.29) | 2.25 (1.89-2.67) | <0.001 | 0.583 | 0.453 |
| V43 | 22.17 (19.13-25.7) | 17.24 (14.87-19.98) | 50.06 (43.19-58.02) | 23.34 (20.14-27.05) | 22.89 (19.75-26.53) | 79.97 (69-92.69) | <0.001 | <0.001 | **<0.001** |
| V44 | 53.67 (3.52-818.29) | 29.49 (1.93-449.61) | 54.66 (3.58-833.4) | 189.47 (12.43-2888.96) | 151.58 (9.94-2311.18) | 2.02 (0.13-30.86) | 0.400 | 0.131 | 0.182 |
| V45 | 200.79 (0.26-154467.7) | 164.11 (0.21-126250.4) | 365.04 (0.47-280822.9) | 190.07 (0.12-113452.4) | 152.19(0.10-325125.1) | 587.79 (0.76-452186.7) | 0.016 | 0.003 | **0.013** |
| V46 | 1.65 (1.2-2.26) | 3.25 (2.37-4.46) | 0.25 (0.18-0.35) | 2.45 (1.78-3.36) | 2.64 (1.92-3.63) | 0.49 (0.36-0.68) | <0.001 | <0.001 | **0.001** |
| V47 | 0.04 (0-0.45) | 7.18 (0.7-73.85) | 9.15 (0.89-94.09) | 5.36 (0.52-55.09) | 0.84 (0.08-8.59) | 0.44 (0.04-4.48) | 0.059 | 0.070 | 0.051 |
| V48 | 10.35 (5.48-19.54) | 6.39 (3.39-12.07) | 23.14 (12.26-43.69) | 1.58 (0.84-2.98) | 1.18 (0.63-2.23) | 38.14 (20.2-72.02) | 0.600 | <0.001 | **0.001** |
| V49 | 0.46 (0.29-0.72) | 0.37 (0.24-0.58) | 0.21 (0.13-0.32) | 2.28 (1.46-3.58) | 4.33 (2.77-6.79) | n.d. | <0.001 | <0.001 | **<0.001** |
| V50 | 0.22 (0-9703.92) | 0 (0-6.87) | n.d. | 0 (0-3.36) | 0.05 (0-2311.26) | n.d. | 0.229 | 0.131 | **0.050** |
| V51 | 0.33 (0.21-0.52) | 0.35 (0.22-0.56) | n.d. | 0.58 (0.36-0.92) | 0.54 (0.34-0.86) | n.d. | <0.001 | <0.001 | **<0.001** |
| *Acids (4)* |  |  |  |  |  |  |  |  |  |
| V52 | 0 (0-1.53) | 0.98 (0-7403.27) | n.d. | 0.74 (0-5549.62) | 0.43 (0-3220.68) | 0.28 (0-2091.43) | 0.252 | 0.029 | 0.210 |
| V53 | 10.76 (7.32-15.83) | 19.17 (13.04-28.18) | 2.66 (1.81-3.91) | 7.52 (5.23-10.16) | 5.31 (2.9-8.2) | 2.76 (1.88-4.06) | <0.001 | <0.001 | **<0.001** |
| V54 | 0.18 (0-92.26) | 1.64 (0-839.25) | 0.32 (0-162.36) | 0.82 (0-262.36) | 0 (0-0.04) | 0.25 (0-128.67) | 0.006 | 0.002 | **0.004** |
| V55 | 4.92 (2.42-9.99) | 7.22 (3.55-14.67) | 2.73 (1.34-5.54) | 0.36 (0.18-0.73) | 1.31 (0.64-2.65) | 10.75 (5.29-21.85) | 0.000 | 0.001 | **0.003** |
| *Pyrazines* (4) | |  |  |  |  |  |  |  |  |
| V56 | 1.09 (0-633.64) | 0.29 (0-168.5) | 0.66 (0-385.1) | 1.34 (0-780.27) | 1.22 (0-707.21) | 0 (0-0.08) | 0.309 | 0.258 | 0.207 |
| V57 | 1.46 (0.76-2.8) | 0.23 (0.12-0.44) | 0.52 (0.27-0.99) | 0.43 (0.22-0.82) | 0.68 (0.36-1.31) | 0.24 (0.12-0.46) | 0.497 | 0.209 | **0.002** |
| V58 | 1.82 (1.59-2.09) | 1.11 (0.97-1.27) | 0.94 (0.82-1.08) | 0.9 (0.79-1.04) | 1.67 (1.46-1.92) | 0.37 (0.32-0.42) | <0.001 | <0.001 | **<0.001** |
| V59 | 2.13 (1.07-4.25) | 0.27 (0.13-0.53) | 0.74 (0.37-1.48) | 0.2 (0.1-0.39) | 0.27 (0.13-0.53) | 0.22 (0.11-0.43) | 0.012 | 0.001 | **0.011** |
| *Others (6)* |  |  |  |  |  |  |  |  |  |
| V60 | 0.68 (0-3240.16) | 0.37 (0-1749.92) | 0.41 (0-1929.72) | 0.61(0-0.129) | 0.71(0-0.231) | 0.18 (0-832.75) | 0.144 | 0.027 | 0.240 |
| V61 | 0.16 (0.11-0.22) | 0.15 (0.1-0.21) | 0.17 (0.12-0.24) | 1.81 (1.28-2.57) | 2.14 (1.51-3.04) | 0.5 (0.35-0.7) | <0.001 | <0.001 | **<0.001** |
| V62 | 0.76 (0.43-1.36) | 0.75 (0.42-1.34) | 0.86 (0.48-1.53) | 0.61 (0.34-1.09) | 0.71 (0.4-1.27) | 1.98 (1.11-3.54) | 0.160 | 0.055 | 0.192 |
| V63 | 2.23 (1.32-3.77) | 3.08 (1.82-5.2) | 0.12 (0.07-0.2) | 0.9 (0.53-1.51) | 1.24 (0.74-2.1) | 0.1 (0.06-0.16) | <0.001 | <0.001 | **0.002** |
| V64 | 3.13 (2.6-3.77) | 2.22 (1.85-2.68) | 1.34 (1.12-1.62) | 2.77 (2.31-3.34) | 2.59 (2.15-3.11) | 1.1 (0.91-1.32) | 0.001 | <0.001 | **<0.001** |
| *V65 | 0.50 | 0.47 | 0.40 | 0.54 | 0.27 | 0.36 | 0.137 | 0.497 | 0.781 |
| n.d.= No detected  Results are presented as back-transformed LSmeans with 95% Confidence intervals (CI) for V1-V64  *=LSmeans with standard error for V65 Phenol, since its data was normally distributed  Analyses were conducted separately for uncooked and cooked samples  LSmeans unit [counts *s * 106] n = 3. | | | | | | | | | |

## A3 Sensory evaluation

### A3.1. Descriptive test

Table A3 Sensory attributes in LSmeans intensities of tempeh prototypes made from faba bean and mix of faba bean and whole-grain oats

| Prototype | 1F  100% faba bean | | 9F  92%Faba bean+8%whole-grain oat | | 8F  85%Faba bean+15%whole-grain oat | | SE | *p-value*, Prototype | *p-value*, LAB | *p-value*, Prototype*LAB |
| --- | --- | --- | --- | --- | --- | --- | --- | --- | --- | --- |
| Soaking | No LAB | LAB | No LAB | LAB | No LAB | LAB |  |  |  |  |
| UNCOOKED | | | | | | | | | | |
| Odour | | | | | | | | | | |
| Overall O. | 5.4^a^ | 5.2^a^ | 5.3^a^ | 5.1^a^ | 5.9^a^ | 5.2^a^ | 0.24 | 0.323 | 0.063 | 0.463 |
| Flour O. | 2.9^a^ | 2.7^a^ | 2.8^a^ | 3.3^a^ | 1.8^b^ | 3.3^a^ | 0.22 | 0.323 | 0.063 | 0.463 |
| Fermented O. | 4.6^a^ | 4.0^a^ | 4.0^a^ | 3.6^a^ | 4.1^a^ | 4.0^a^ | 0.27 | 0.233 | 0.135 | 0.595 |
| Beany O. | 3.5^b^ | 3.0^b^ | 2.9^b^ | 3.2^b^ | 2.5^a^ | 3.3^b^ | 0.20 | 0.250 | 0.244 | **0.029** |
| Butter O. | 2.5^a^ | 2.4^a^ | 2.4^a^ | 2.5^a^ | 2.8^a^ | 2.8^a^ | 0.25 | 0.358 | 0.963 | 0.880 |
| Nutty O. | 2.3^b^ | 2.4^b^ | 3.0^b^ | 3.3^b^ | 1.9^c^ | 3.8^a^ | 0.28 | **0.038** | **0.009** | **0.015** |
| Sweet O. | 2.9^c^ | 2.9^c^ | 3.5^a^ | 3.2^b^ | 3.6^a^ | 3.3^b^ | 0.17 | **0.019** | 0.186 | 0.587 |
| Ethanol O. | 2.3^c^ | 2.9^b^ | 2.9^b^ | 2.4^b^ | 4.7^a^ | 2.4^b^ | 0.27 | **0.008** | **0.006** | **<0.001** |
| Musty O. | 3.1^a^ | 2.4^a^ | 2.9^a^ | 2.9^a^ | 2.5^a^ | 2.8^a^ | 1.18 | 0.770 | 0.638 | 0.414 |
| COOKED | | | | | | | | | | |
| Odour | | | | | | | | | | |
| Overall O. | 5.0^a^ | 5.4^a^ | 5.7^a^ | 5.9^a^ | 5.7^a^ | 5.7^a^ | 0.30 | 0.137 | 0.497 | 0.781 |
| Flour O. | 3.6^a^ | 3.4 ^a^ | 3.5^a^ | 3.8^a^ | 3.3^a^ | 4.3^a^ | 0.30 | 0.676 | 0.150 | 0.212 |
| Fermented O. | 3.1^a^ | 3.6^a^ | 3.4^a^ | 3.2^a^ | 4.1^a^ | 3.5^a^ | 0.38 | 0.381 | 0.689 | 0.362 |
| Beany O. | 3.5^a^ | 3.6^a^ | 3.7^a^ | 3.8^a^ | 3.2^a^ | 4.0^a^ | 0.33 | 0.876 | 0.222 | 0.487 |
| Butter O. | 2.0^b^ | 1.7^c^ | 2.5^b^ | 2.4^b^ | 2.0^b^ | 2.7^a^ | 0.21 | **0.022** | 0.440 | 0.060 |
| Nutty O. | 2.8^d^ | 3.4^c^ | 3.7^b^ | 4.0^a^ | 3.0^d^ | 3.7^b^ | 0.23 | **0.033** | **0.017** | 0.593 |
| Sweet O. | 2.2^b^ | 2.4^b^ | 2.9^b^ | 2.7^b^ | 3.4^a^ | 2.8^b^ | 0.23 | **0.015** | 0.299 | 0.233 |
| Ethanol O. | 1.4^b^ | 1.5^b^ | 1.9^c^ | 1.6^bc^ | 2.8^a^ | 1.6^bc^ | 0.22 | **0.015** | **0.025** | **0.035** |
| Musty O. | 3.3^a^ | 2.8^a^ | 2.7^a^ | 3.0^a^ | 2.8^a^ | 3.2^a^ | 0.37 | 0.899 | 0.734 | 0.430 |
| Taste |  |  |  |  |  |  |  |  |  |  |
| Sweetness | 1.4^a^ | 1.8^a^ | 1.5^a^ | 1.9^a^ | 1.8^a^ | 1.6^a^ | 0.21 | 0.869 | 0.299 | 0.271 |
| Sourness | 3.2^c^ | 3.6^bc^ | 3.3^c^ | 3.7^b^ | 4.5^a^ | 4.1^a^ | 0.25 | **0.006** | 0.436 | 0.151 |
| Bitterness | 3.2^a^ | 3.7^a^ | 3.4^a^ | 3.4^a^ | 3.6^a^ | 3.6^a^ | 0.38 | 0.841 | 0.657 | 0.740 |
| Astringency | 3.2^a^ | 3.1^a^ | 3.0^a^ | 3.3^a^ | 3.6^a^ | 3.2^a^ | 0.26 | 0.672 | 0.763 | 0.424 |
| Umami | 3.8^c^ | 4.5^b^ | 3.6^c^ | 4.6^b^ | 3.7^c^ | 5.1^a^ | 0.30 | 0.624 | **0.001** | 0.501 |
| Texture |  |  |  |  |  |  |  |  |  |  |
| Graininess | 4.7^a^ | 4.0^cd^ | 4.5^ab^ | 3.5^d^ | 4.4^bc^ | 3.6^cd^ | 0.25 | 0.326 | **0.001** | 0.908 |
| Crumbliness | 5.2^a^ | 4.1 | 4.4 | 3.5 | 4.3 | 2.9 | 0.30 | **0.015** | **<0.001** | 0.669 |
| Chewiness | 3.5^d^ | 4.4^c^ | 5.0^b^ | 6.5^a^ | 4.4^c^ | 6.3^a^ | 0.35 | **0.001** | **<0.001** | 0.438 |
| Softness | 6.5^a^ | 5.9^b^ | 6.0^b^ | 4.5^d^ | 6.3^a^ | 5.2^c^ | 0.32 | **0.040** | **0.002** | 0.383 |
| Appearance |  |  |  |  |  |  |  |  |  |  |
| Golden C. | 3.3^d^ | 4.5^b^ | 4.8^b^ | 3.6^c^ | 5.7^a^ | 3.8^c^ | 0.40 | 0.146 | 0.081 | **0.004** |
| Grey C. | 6.0^a^ | 4.3^c^ | 4.4^c^ | 5.6^b^ | 2.8^d^ | 5.3^b^ | 0.44 | 0.065 | 0.115 | **0.001** |
| Compactness | 3.5^d^ | 5.6^b^ | 5.8^b^ | 6.5^a^ | 5.2^c^ | 6.0^a^ | 0.36 | **0.002** | **0.001** | 0.117 |
| O.= Odours  Values represent LSmeans and SE.  Mean values in the same row with different letters differ significantly (*p < 0.05*)  Bold *p-values* represent significant differences  n = 24  Scale 0 to 10, 0=absence and 10= max intensity | | | | | | | | | | |

### A3.2. Questionnaire form

#### Uncooked

Example of the questionnaire to investigate and construct the aroma profile based on the odours quantification of uncooked samples.
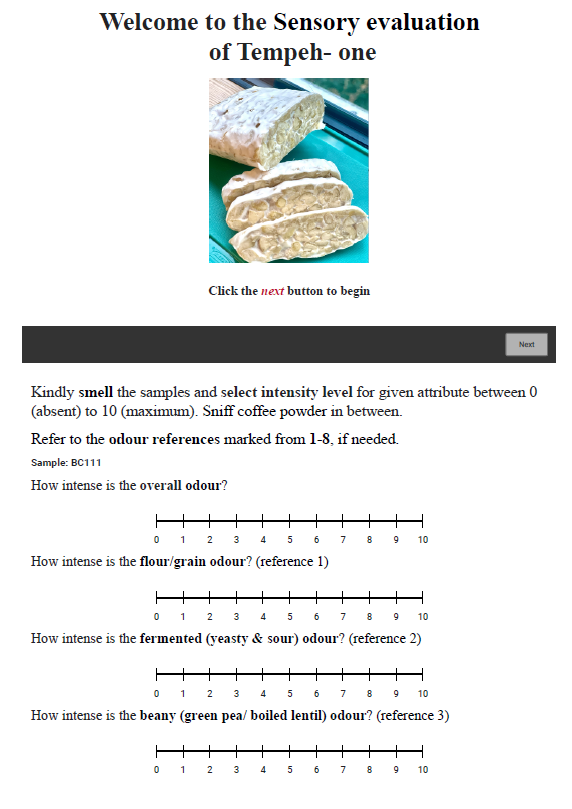


#### Cooked

Example of the questionnaire to investigate and construct the sensory profile based on the odours, taste, texture and appearance quantification of cooked samples.


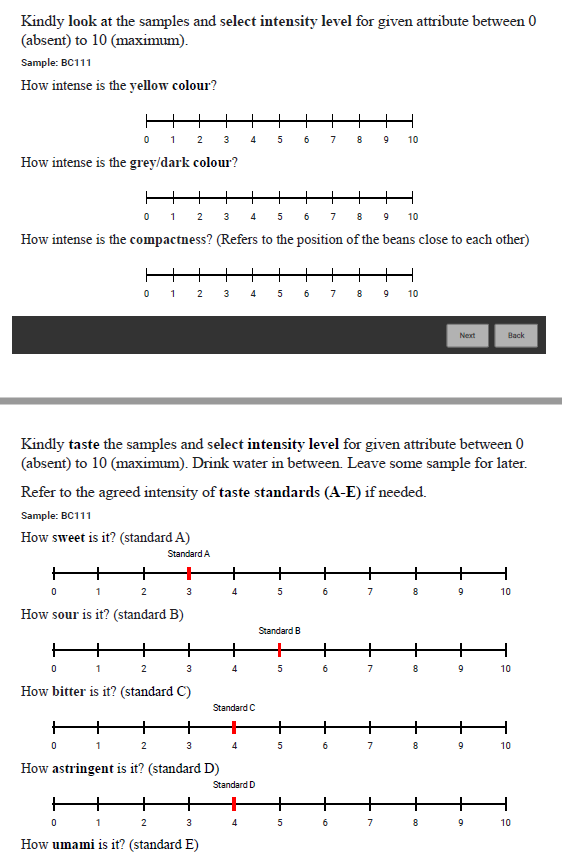


### A3.3. Hedonic test


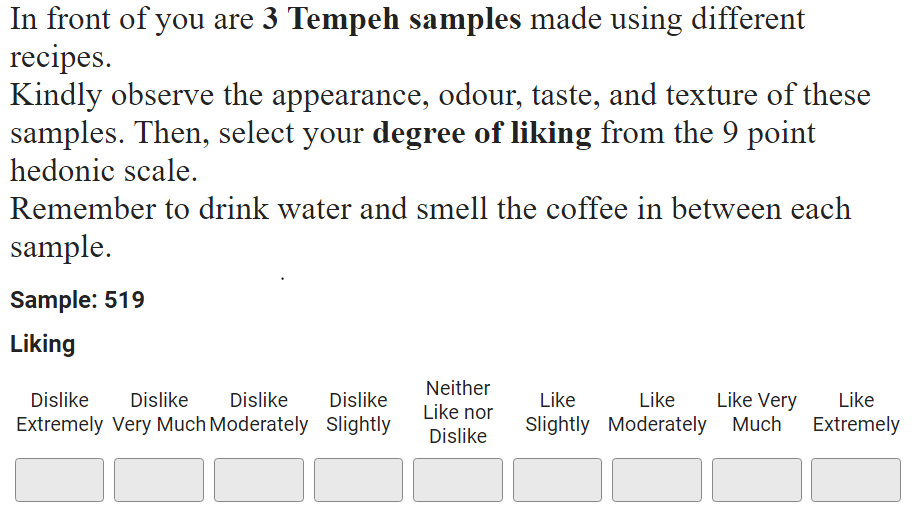


## A4. Samples


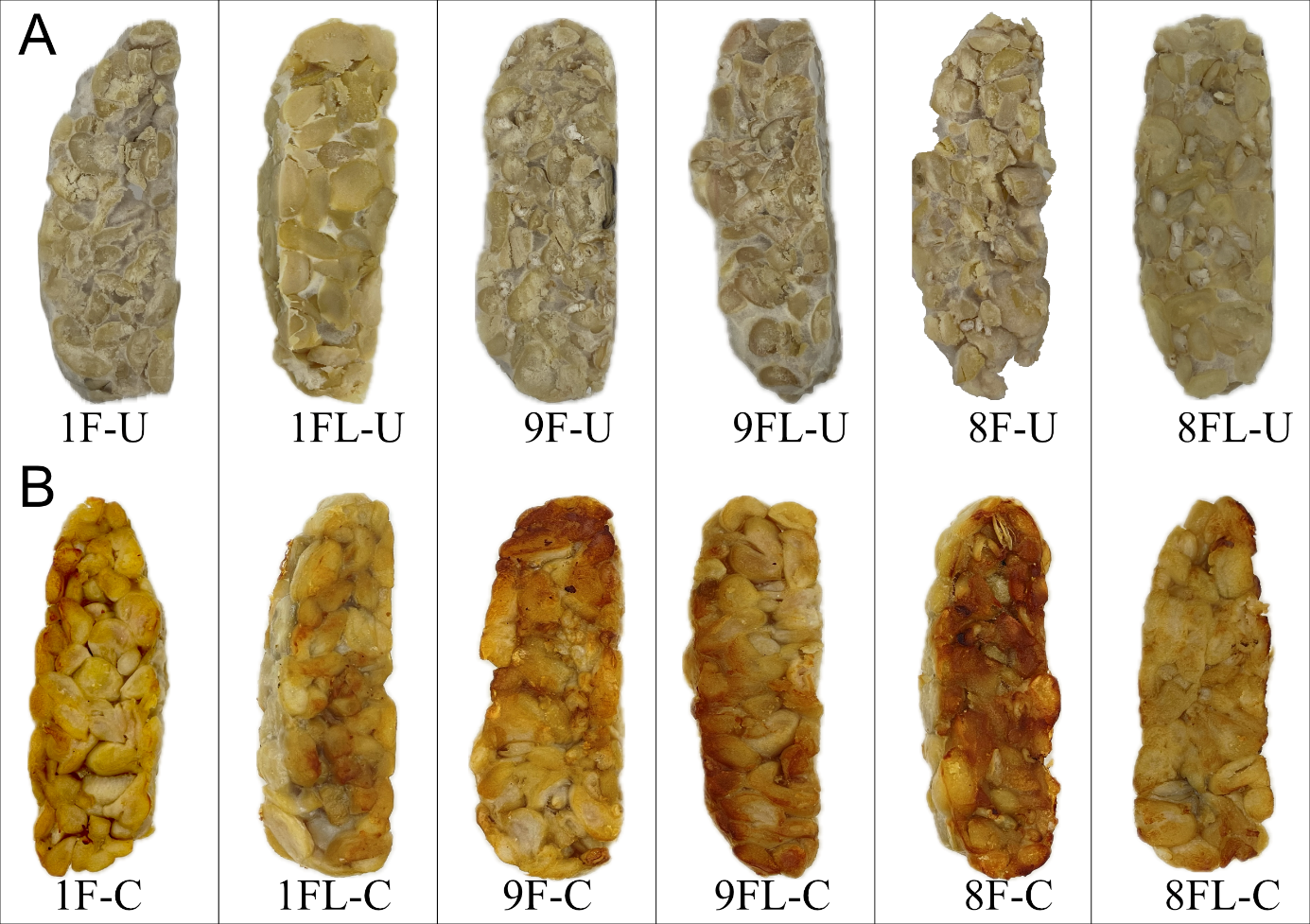


Figure 1 Picture of tempeh prototypes corresponding to 1F= 100% faba bean, 9F= 92% faba bean + 8% whole-grain oat and 8F= 85% Faba bean + 15% whole-grain oat without and with L=*L. plantarum*. –U= Uncooked and –C= cooked. All cooked samples were roasted in a frying pan for 2 min per side.
